# Alternative futures for Borneo show the value of integrating economic and conservation targets across borders

**DOI:** 10.1038/ncomms7819

**Published:** 2015-04-14

**Authors:** Rebecca K. Runting, Erik Meijaard, Nicola K. Abram, Jessie A. Wells, David L.A. Gaveau, Marc Ancrenaz, Hugh P. Posssingham, Serge A. Wich, Fitrian Ardiansyah, Melvin T. Gumal, Laurentius N. Ambu, Kerrie A. Wilson

**Affiliations:** 1 Australian Research Council Centre of Excellence for Environmental Decisions, School of Biological Sciences, The University of Queensland, Brisbane 4072, Queensland, Australia; 2 Borneo Futures Project, Country Woods 306, Jalan WR Supratman, Pondok Ranji-Rengas, Ciputat 15412, Tangerang, Indonesia; 3 School of Geography, Planning and Environmental Management, The University of Queensland, Brisbane 4072, Queensland, Australia; 4 Center for International Forestry Research, P.O. Box 0113 BOCBD, Bogor 16000, Indonesia; 5 Durrell Institute for Conservation and Ecology, School of Anthropology and Conservation, Marlowe Building, University of Kent, Canterbury, Kent CT2 7NS, UK; 6 HUTAN/Kinabatangan Orangutan Conservation Programme, PO Box 3109, 90734 Sandakan, Sabah, Malaysia; 7 Living Landscape Alliance, 5 Jupiter House, Calleva Park, Aldermaston, Reading RG7 8NN, UK; 8 Sabah Wildlife Department, Wisma MUIS, 88100 Kota Kinabalu, Sabah, Malaysia; 9 North England Zoological Society, Upton-by-Chester, Chester CH2 1LH, UK; 10 School of Life Sciences, Imperial College, Silwood Park Campus, Berkshire, SL5 7PY UK; 11 School of Natural Sciences & Psychology, Liverpool John Moores University, Hatton Garden, Liverpool L3 2AJ, UK; 12 Institute for Biodiversity and Ecosystem Dynamics, University of Amsterdam, Science Park 904, 1098 XH Amsterdam, The Netherlands; 13 Crawford School of Public Policy, The Australian National University, Canberra, 0200 ACT, Australia; 14 Pelangi Indonesia, Jl. Masjid III No. 25, Pejompongan, Jakarta 10210, Indonesia; 15 IDH-The Sustainable Trade Initiative, , Nieuwekade 9, 3511 RV Utrecht, The Netherlands; 16 Wildlife Conservation Society, Malaysia Program, 7, Jalan Ridgeway, 93200 Kuching, Sarawak, Malaysia

## Abstract

Balancing economic development with international commitments to protect biodiversity is a global challenge. Achieving this balance requires an understanding of the possible consequences of alternative future scenarios for a range of stakeholders. We employ an integrated economic and environmental planning approach to evaluate four alternative futures for the mega-diverse island of Borneo. We show what could be achieved if the three national jurisdictions of Borneo coordinate efforts to achieve their public policy targets and allow a partial reallocation of planned land uses. We reveal the potential for Borneo to simultaneously retain ∼50% of its land as forests, protect adequate habitat for the Bornean orangutan (*Pongo pygmaeus*) and Bornean elephant (*Elephas maximus borneensis*), and achieve an opportunity cost saving of over US$43 billion. Such coordination would depend on enhanced information sharing and reforms to land-use planning, which could be supported by the increasingly international nature of economies and conservation efforts.

## Abstract (web summary)

Balancing biological conservation with economic development is a challenge for policymakers. Analysing a range of possible future scenarios, Runting *et al*, show that coordinated planning would allow the island of Borneo to simultaneously retain forested land, protect species and meet economic goals.

## Graphical abstract

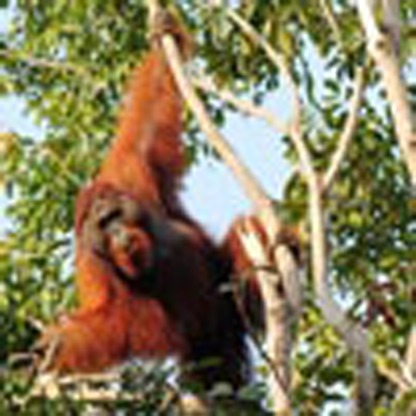

All United Nations member states have sanctioned national efforts to pursue environmental sustainability under the Convention on Biological Diversity and the Millennium Development Goals. Simultaneously, states have set ambitious national targets for economic growth, development and trade, often without assessing how these targets align or conflict with sustainability agendas. Balancing the needs for economic development with international commitments to protect biodiversity is a global challenge. Achieving this balance will require a whole-landscape approach to land-use planning that incorporates the targets sought by multiple sectors^1^. The potential for systematic planning approaches to deliver large gains in economic and environmental efficiency has so far been demonstrated in efforts to re-design protected area networks within^2^ and across^3^ political borders. We now need to understand whether this potential can be realised in regions with multiple land uses and multiple, often conflicting, objectives. Sustainable allocation of land uses will require a dialogue on potential futures and an understanding of the possible consequences of alternative strategies for diverse sectors^4^,^5^.

Tropical forests regulate regional and global climate, provide a wide range of ecosystem services to over a billion people, and support ∼50% of described species^6^,^7^,^8^. The forests of Borneo, the third largest island in the world, have an average above-ground biomass that is 60% higher than the Amazonian average^9^. The island harbours an estimated 14,423 plant and 1,640 vertebrate species, of which 28% are endemic (Supplementary Table 1) and 534 (3%) are considered to be threatened with extinction^10^. The extent of forest on Borneo declined by 16.8 × 10^6^ ha (30%) from 1973–2010 because of agricultural expansion and ENSO-induced wildfires^11^. Indonesia and Malaysia are major exporters of palm oil; in 2012 these countries collectively produced >80% of the global supply^12^. Furthermore, the governments of Malaysia and Indonesia seek to increase the area of oil palm and industrial timber plantations (ITPs) on Borneo by 7.1 × 10^6^ ha over the next two decades. The planned expansion of oil palm plantations in Indonesian Borneo alone is projected to contribute carbon dioxide emissions (CO_2_) of 0.12–0.15 GtC yr^−1^ from 2010 to 2020, equating to ∼34% of Indonesia's total land sector emissions^13^. High rates of forest conversion and degradation have prompted inter-governmental agreements between Indonesia, Malaysia and Brunei Darussalam to protect and sustainably use the forests that remain in Borneo^14^. For example, the Borneo Initiative is a project focused on sustainable forest management^15^, and the Heart of Borneo initiative aims to sustainably manage ∼20 × 10^6^ ha of the mountainous core of the island^16^. While political coordination across borders will likely improve the efficiency of meeting economic and conservation goals, these potential gains have not previously been quantified.

We explored four alternative futures for Borneo, each representing a set of policy objectives and a planning strategy: (1) baseline (current land-use allocations are executed); (2) uncoordinated, state-based planning to achieve policy targets (with the Malaysian states of Sabah and Sarawak treated separately); (3) coordinated planning in the mountainous interior of Borneo, with state-based planning outside this area; and (4) integrated planning across all four states (allowing for both jurisdictional coordination and the reallocation of some land uses) to achieve either (a) existing public policy targets or (b) alternative biodiversity targets seeking to achieve representative protection of dominant vegetation types (Table 1). For each scenario (except the baseline), we identified land-use configurations that achieve the stated targets. We evaluated each scenario by determining the opportunity costs of meeting existing policy targets for key economic and conservation features, namely forest cover, protected areas, Bornean orangutan (*Pongo pygmaeus*), Bornean elephant (*Elephas maximus borneensis*), oil palm and ITP (Table 2). We also evaluated the scenarios in terms of the extent of land allocated to conventional (CL) or reduced-impact logging (RIL) and the potential for reducing CO_2_ emissions relative to the baseline scenario. We reveal the potential for Borneo to simultaneously: retain ∼50% of its land as forests, protect adequate habitat for orangutan and elephant, and achieve an opportunity cost saving of over US$43 billion. The value of integrating economic and conservation goals through trans-boundary collaboration will be substantial wherever the costs and opportunities for achieving goals vary across borders.

## Results

### Protecting the mountainous interior of Borneo

**Figure 1:**
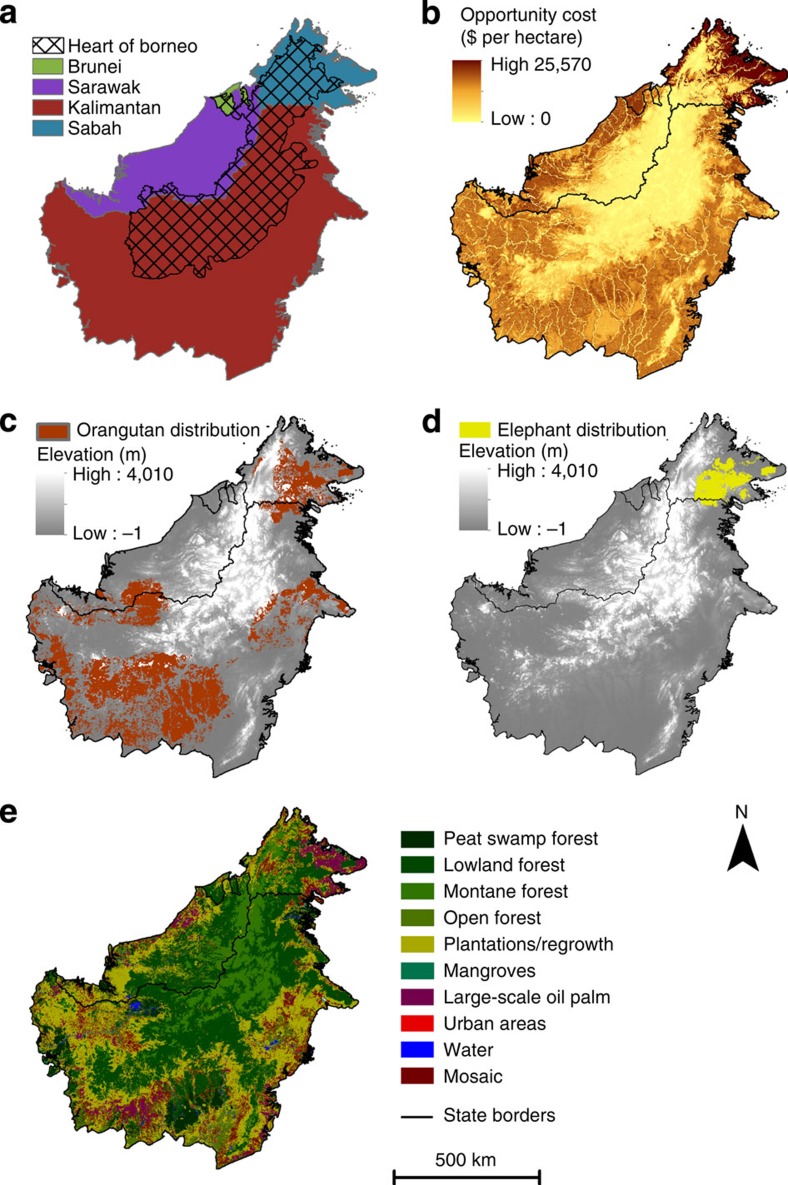
Context of Borneo. (**a**) Bornean states and the planned area for the Heart of Borneo initiative. (**b**) The opportunity cost (per hectare) of designating land as ‘Protected'. An opportunity cost layer was developed separately for each of the possible land uses. (**c**,**d**) The distribution of orangutan and elephant, respectively. (**e**) Current land use and land cover^63^. The orangutan distribution map is based on a predictive model^54^, and is continually updated as new information becomes available on the presence and absence of the species from different regions. For example, we note that in 2015–2016 additional surveys in Sarawak will be carried out by the Wildlife Conservation Society.

**Figure 2:**
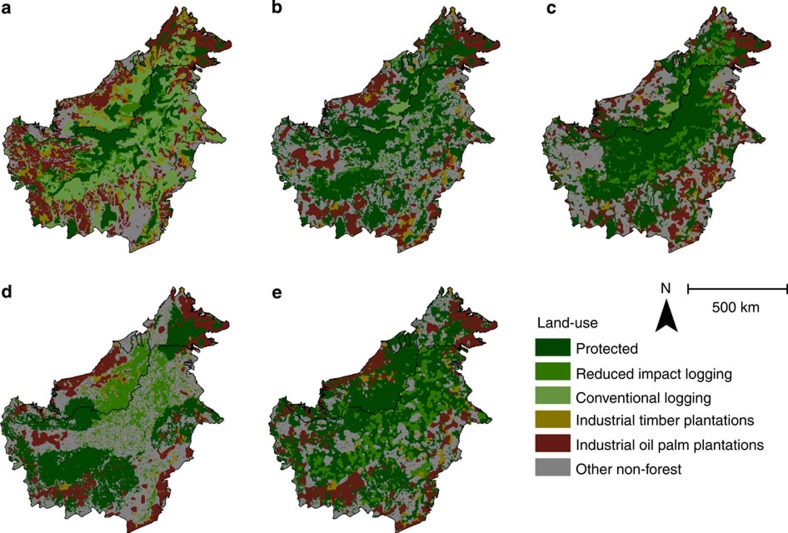
Future land-use options under each scenario: (**a**) baseline (scenario 1); (**b**) state-based planning (scenario 2); (**c**) coordinated planning within the mountainous core, with state-based planning outside (scenario 3); (**d**) integrated planning with existing state targets (scenario 4a); and (**e**) integrated planning with alternative public policy targets for biodiversity (scenario 4b).

**Figure 3:**
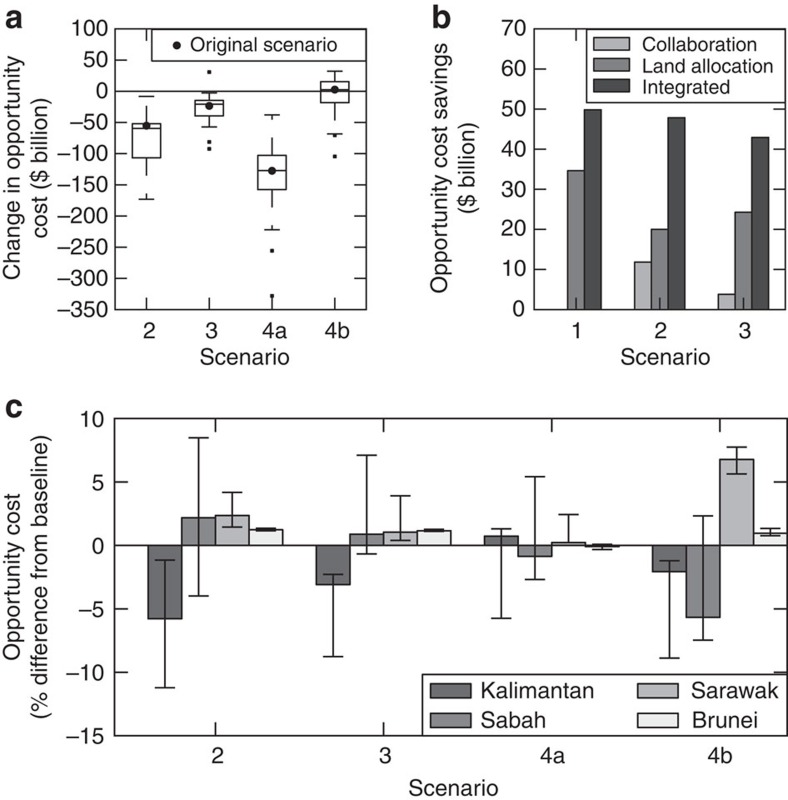
Changes in opportunity costs under the alternative planning scenarios. (**a**) Comparing opportunity costs relative to the baseline (scenario 1), integrated planning (scenario 4a) resulted in the lowest opportunity cost, whereas extending the conservation targets (scenario 4b) was most expensive. Box plots show the variation in opportunity costs when altering the economic parameters and assumptions about public policy targets. While this variation was considerable, it affected all scenarios similarly, such that integrated planning had the lowest opportunity cost for any given set of parameters and assumptions. (**b**) Exploring the effects of coordination and/or allowing more flexible changes to existing land allocations on the opportunity cost for scenarios 1, 2 and 3. Savings are expressed relative to the opportunity cost of each scenario when it is implemented without full coordination, and allowing fewer changes to the existing land allocation. (**c**) The distribution of opportunity cost among states differed in each scenario, compared with the baseline case (scenario 1). Although each state's opportunity cost differed by a maximum of ±7% between scenarios, this is still likely to create challenges for collaborative efforts. The error bars represent the minimum and maximum opportunity cost change when altering the economic parameters and assumptions about public policy targets.

**Figure 4:**
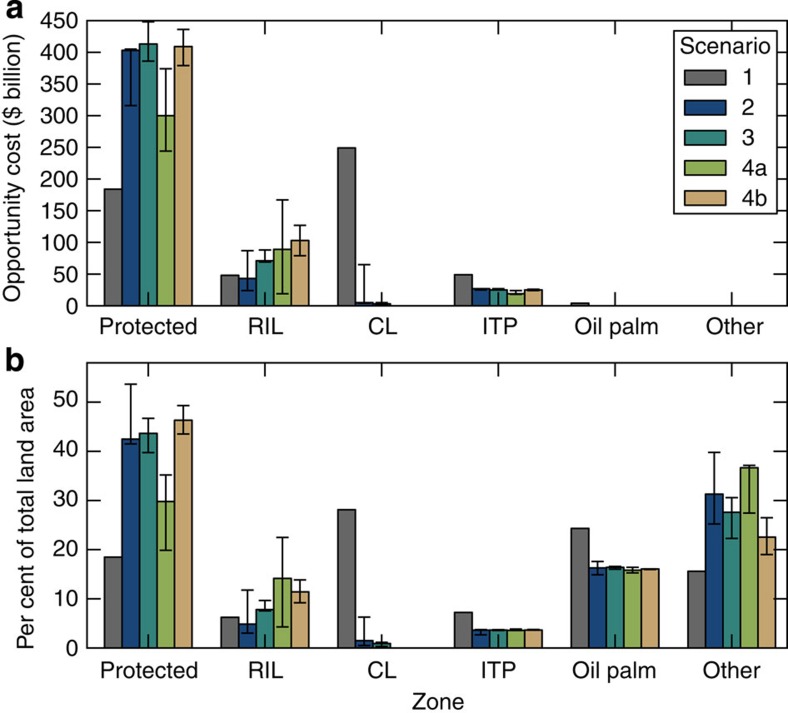
Allocation of land uses across scenarios. (**a**) The contribution of each land-use zone to the opportunity cost. (**b**) The per cent of total land area allocated to each land use under alternative scenarios. CL and RIL refer to conventional logging and reduced-impact logging, respectively. ITP refers to industrial timber plantations. Solid bars represent the result from each scenario, and the error bars represent the minimum and maximum when altering the economic parameters and assumptions about public policy targets. The baseline (scenario 1) shows no variation, as it assessed the existing land-use allocations.

The aspirations of the highest profile conservation initiative in Borneo (the Heart of Borneo) are reflected in scenario 3, with coordinated efforts focused on the mountainous and heavily forested interior of Borneo, and state-based planning outside of this core region (Figs 1a and 2c). This scenario incurs the greatest opportunity cost for meeting the policy targets, as 51% of land on Borneo would be required for protection or reduced-impact logging (Figs 3a and 4). While large tracts of land remain forested under this scenario, much of the lowland habitat for orangutan and elephant is converted to non-forest use, as these areas fall outside of the core region and existing protected areas (Figs 1 and 2). Despite these limitations, this scenario substantially improves on conservation targets relative to the baseline scenario (scenario 1), which could result in only 25% of land protected or managed for reduced-impact logging and the remainder being converted to non-forest use or conventional forestry (Fig. 4b).

### Integrated planning achieves targets more efficiently

**Figure 5:**
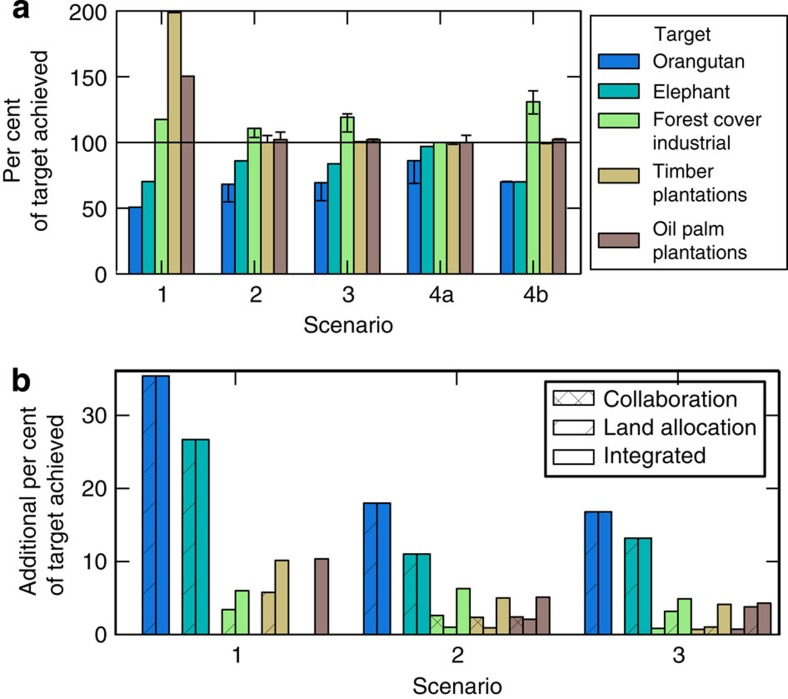
Variation between scenarios in terms of their achievement of public policy targets. (**a**) All scenarios achieved the economic targets (that is, industrial timber plantations and oil palm plantations), but no scenarios achieved the species conservation targets. Integrated planning (scenario 4a) performed the best in terms of minimizing the overall target shortfall. The target for protected areas is not shown, because the target of 17% by land area was met in the baseline scenario, and was greatly exceeded in scenarios 2, 3 and 4 due to the orangutan and elephant habitat requirements. The error bars represent the minimum and maximum change in target achievement when altering the economic parameters and assumptions about public policy targets. (**b**) More of the species conservation targets can be achieved when planning involves coordination between Bornean states, and/or allowing more flexible changes to existing land allocations. Allowing more flexible changes to existing land allocations resulted in substantial gains for species conservation targets because much of the orangutan and elephant habitat overlaps with unplanted concessions for industrial timber or oil palm. Allowing these areas to become protected or logged forests markedly increases the scope for achieving the targets for these threatened species.

Integrated planning both within individual states and across jurisdictional borders could enable substantial savings while meeting targets across diverse sectors. If states coordinated their plans and allowed more flexible changes to existing land-use allocations (scenario 4a, Supplementary Fig. 1), this would offer an opportunity cost saving of at least US$43 billion with the same level of target achievement as other scenarios (Fig. 3b), or, for a similar opportunity cost, would enable substantially higher achievement of all targets (Fig. 5). In addition, integrated planning was the closest to meeting conservation targets while requiring less land for protected areas, and delivering the greatest area of reduced-impact logging (Figs 5 and 4b).

A shift away from state- or species-focused approaches to a more collaborative, ecosystem-based approach could deliver substantial dividends for climate change mitigation and for biodiversity conservation. Integrated planning reduces CO_2_ emissions from land-use change relative to the baseline, and out-performs other scenarios if the forest cover target is modified from a target for total forest cover (regardless of forest type), to a target of conserving 70% of the remaining extent of each forest type (scenario 4b, Supplementary Fig. 2). With a ‘total forest' target (scenario 4a), protected areas are concentrated within the remaining extent of orangutan and elephant distributions, with limited protection of upland forests (Figs 1 and 2, Supplementary Fig. 3), and emission reductions are ∼16%. In contrast, if forest cover targets require conservation of each forest type (scenario 4b), then it is possible to achieve a 53% reduction in emissions compared with the baseline (Supplementary Fig. 2). This scenario therefore offers emission reductions that are substantially higher (53 versus 40%) than would be possible if protection was concentrated in the mountainous core of the island (scenario 3), even though opportunity costs remain similar.

### Integrated planning requires some reassignment of land uses

Our alternative futures reveal that public policy targets can be more efficiently achieved through coordination and modifications to existing land-use allocations. Integrated planning across Borneo (scenario 4a) could require protection of 8.6 × 10^6^ ha of land that is currently designated for logging (with or without an existing concession), along with 4.3 × 10^6^ ha of unplanted oil palm concessions and 1.3 × 10^6^ ha of unplanted industrial timber concessions (Supplementary Fig. 4). Despite this substantial reallocation of land uses, the opportunity costs to each state remained similar to the baseline scenario (each state's opportunity costs differed by a maximum of ±7% across all scenarios; Fig. 3c). Nonetheless, even small differences in opportunity costs may create challenges for collaboration. There are also some substantial differences across states in the land allocations required to meet targets (even if total opportunity costs are similar). For example, in scenario 4b, the extent of protected areas is increased by 58% (compared with baseline) in Sarawak, compared with 20% in Kalimantan and 14% in Sabah, which partly reflects their existing protected area estate, and differences across states in opportunity costs of logging and plantations (Supplementary Fig. 5).

The allocation of land uses within each of the scenarios changed with variation in parameter values and multiple model runs (Supplementary Fig. 6). While the spatial allocation of protected areas and RIL varied only slightly (reflecting the limited spatial ranges and habitat requirements of orangutan and elephant), the allocation of the other land uses was relatively flexible, reflecting the much greater availability of land suitable for oil palm and ITP. This flexibility in the allocation of land to oil palm and ITP means that the land-use scenarios presented here (Fig. 2) could be adjusted to accommodate local needs without compromising overall economic targets.

## Discussion

Integrated land-use planning has the potential to achieve a wide range of targets in a cost-effective manner, but the effectiveness of any planning process also depends critically on the adequacy of public policy targets. For example, the integrated planning scenario (scenario 4a) would cost-effectively make progress towards the stated species conservation targets (Fig. 3a), but the allocation of protected areas would be biased towards habitat favoured by orangutan and elephant (Figs 1c,d, 2d) and potentially at the expense of other species or the livelihoods of local people^17^. While ignoring existing targets could lead to substantial savings (Supplementary Fig. 7), it could result in poor conservation outcomes (Supplementary Fig. 8). In contrast, if targets existed for each major vegetation type (scenario 4b) then greater geographic representation of the various habitats would be ensured (Supplementary Fig. 3), and this would also substantially enhance opportunities to reduce CO_2_ emissions from land-use change (Supplementary Fig. 2). To facilitate integrated planning, Borneo-wide targets would need to be fully backed by all of the governments of Borneo, be developed in the context of other aligned or potentially conflicting goals, and respect political and economic sovereignty. This issue is not unique to Borneo—developing quantifiable targets to achieve ecologically sustainable development is a global challenge^18^.

Given the vast spatial extent of Borneo and the multitude of factors included in this analysis, we acknowledge that the data and assumptions will not capture local variation and nuances, particularly in relation to opportunity costs. We have not, for example, accounted for the potential that one land-use type might have a greater rate of change in profitability over time, or that the spatially explicit probability of conversion might change over time. Furthermore, a fully functioning market for carbon would likely reduce the relative opportunity costs of the scenarios that offer higher emission reductions. However, we found that large variations in input parameters (including alternative interpretations of public policy targets) would not change the overall conclusions (Supplementary Table 2). We have also not attempted to analyse all potential futures, but rather we reveal the possible outcomes of an illustrative set of planning options.

We found that changing the status of unplanted oil palm and industrial timber concessions will be vital for making progress towards species conservation targets (Fig. 5). We acknowledge that reallocating undeveloped land would not be trivial, and will require a thorough evaluation of tenure and governance arrangements in all stages of the planning process^19^. Careful consideration of the appropriate institutional and incentive structures will be vital and require consultation beyond state and inter-governmental bodies to include the business sector, local communities and the wider public. To realize conservation and economic goals on the ground, institutional arrangements would also need to ensure that incentives reach key actors at a district or local level^20^.

Implementing an integrated planning approach (scenario 4a and 4b) requires both new protected areas to be designated and managed, and also for some existing protected areas to be reallocated to other land uses (Supplementary Fig. 4). This process of protected area downgrading, downsizing or degazettement (PADDD) may risk undermining the perceived permanence of other protected areas^21^. Despite this issue, PADDD may be an essential part of land-use planning reform and substantial efficiency gains and improved biodiversity outcomes could be achieved by re-allocating underperforming protected areas^2^. Globally, protected areas are biased towards areas that have limited development potential (such as remote areas, or those with steep slopes or high elevation)^22^. This is also true with Borneo, where protected areas are concentrated in the mountainous interior, resulting in a biased representation of forest types (that is, montane forests above all other types, Supplementary Fig. 3). In other locations the effectiveness of protected areas is reduced by surrounding land uses^11^. Laurance *et al*,^23^ found that half of protected areas in the world's tropical forests are ineffectively managed, resulting in a loss of biodiversity—a process that was strongly influenced by the surrounding landscape. Reallocating protected areas within the context of whole-landscape land-use planning may outweigh the risks associated with PADDD. However, a broader range of conservation targets must be developed and assessed before determining the optimal allocation of protected areas.

The capacity to effectively implement public policy targets varies significantly among the geopolitical units of Borneo^24^. Trans-national coordination would need to overcome constraints related to governance efficacy, efficiency, regulatory quality, sovereignty commitments and control of corruption^25^. Furthermore, the history of cooperation between Brunei Darussalam, Malaysia and Indonesia has involved significant challenges^26^,^27^. Substantial complexity is added by sectorial control of different land-use types (for example, forestry, agriculture and mining), the related political territoriality and by varying social acceptability of land-use changes^28^. A socially equitable distribution of land use might be well received by local communities, but deriving such a land-use plan will require quantification of institutional and individual costs and constraints not yet captured in our analysis. Innovative mechanisms, such as land swaps and payments for conservation or opportunities foregone between geopolitical units (states, provinces and districts) may be required for the direct and indirect benefits of integrated planning to be realised^29^.

Our results confirm that there is a strong justification for expanding existing efforts for collaboration across the political borders of Borneo. This finding is in line with Kremen *et al*,^30^, who found that operating at the national scale was ineffective in achieving conservation outcomes. Our study has demonstrated that restricting coordination to within the mountainous interior (that is, the Heart of Borneo, scenario 3) fails to realize the benefits of wider coordination and will not meet public policy targets. While the Heart of Borneo initiative reflects the sentiment of coordinated planning, stronger and more geographically distributed efforts are needed to avoid irreversible biodiversity loss, achieve equitable benefits among diverse stakeholders, and maximize efficiency across multiple sectors. A binding agreement on land use may be necessary to ensure that jointly developed plans are implemented in each national jurisdiction. Such an agreement could be facilitated by a regional inter-governmental platform (such as ASEAN (The Association of Southeast Asian Nations), the tri-national collaboration regarding the Heart of Borneo or BIMP-EAGA (Brunei Darussalam–Indonesia–Malaysia–Philippines East ASEAN Growth Area)) and should serve to give each jurisdiction the confidence that their interests are being treated equitably. The agreement could include joint targets for sustainable management of forests, facilitate technical exchange on how to achieve these targets, bring cross-border protected areas under joint management, and address cross-border trade and flow of labour. While designing such an agreement will involve many challenges, a non-binding agreement risks weak implementation and the adverse environmental impacts from poorly regulated agricultural expansion and extractive industries^31^.

Our study is based on the fundamental assumption that governments seek to achieve their stated public policy targets, and that all targets are weighted equally. The reality, however, is that there will be far greater governmental support for increasing profits from oil palm and other lucrative activities, as opposed to meeting conservation targets (for example, the Indonesian government's target to stabilize all wild orangutan populations by 2017)^32^. This situation is reinforced by the close and well-protected ties between industry (for example, oil palm, forestry, mining and so on), and politicians^33^,^34^; the intertwining relationships between, rather than independence of, the executive, legislative and judicial branches of government^35^; and corruption in both Indonesia and Malaysia^36^,^37^. Opposing these barriers, however, are potentially powerful democratic forces, such as the growth of local non-government organizations and the relative freedom of speech and information, especially in Indonesia^38^. Access to information is an important precursor to change in political and civil society, including the potential for policy reform and implementation of innovative solutions^35^.

All countries on Borneo are struggling to develop and implement strategies that achieve sustainability despite their stated commitments to green growth and sustainable development. For example, the Sabah government has committed to certifying all its remaining natural forest timber concessions under the criteria of the Forest Stewardship Council or the Malaysian Timber Certification Council (Table 2, Supplementary Table 3). However, the over-logged forests in Sabah raise limited net revenue, requiring the operations to be scaled back until forests have sufficiently recovered to once again produce commercial timber^39^. Alternatively, authorities could potentially generate income from avoided deforestation (requiring the development of a regulatory framework that aligns with international criteria for carbon trade), or from intensification of plantation production. The latter would require new spatial plans that allow plantation development within commercial forest reserves, along with stringent safeguards to minimize impacts on other targets (for example, targets included in the State action plans for elephant and orangutan, the Sabah Biodiversity Strategy (2012–2022), Sabah Tourism Masterplan (2011–2025) and the Sabah Structural Plan (2013–2033)). It may also be necessary to alter existing legislation, which can require landholders to clear any forest on their land within a specified time period (usually 3 years)^40^. Certification through the Roundtable on Sustainable Palm Oil (RSPO) has the potential to minimize adverse environmental impacts from oil palm expansion, but significant high-level reforms to its monitoring, enforcement and auditing processes are needed for this to be an effective option^41^. Obstacles such as these have to be overcome before the benefits of land-use policy reform can be realised.

New mechanisms are required to ensure effective implementation of the targets evaluated here. In some districts, for example, targets for watershed management or wildlife conservation will require new or expanded protected areas. Under such circumstances, a payment scheme to reward districts (or states or countries) for delivering these goods and services may incentivize protection. Payments for environmental service schemes have been piloted in Indonesia^42^ but have primarily been initiated by private enterprise. A regulatory framework to facilitate payments between districts is being drafted under the government regulation on environmental management, but is still awaiting endorsement^43^. A broader regulatory and institutional framework that encompasses such schemes and new market-based mechanisms will be essential to deliver effective land-use planning and land management.

The potential benefits from integrated planning within and between countries are not unique to the island of Borneo; many other jurisdictions across the globe have committed to land-use allocations that are proving to be suboptimal. For example, Australia has devoted over half of its land mass to low productivity pastoralism with inflexible leasehold arrangements^44^, and China's farmland protection policy has led to a clustering of incompatible land uses^45^. Trans-national collaboration may also be beneficial in the Congo Basin—a globally significant forest area spanning six central African countries with varying deforestation rates, with competing potential uses of the forest area^46^. Such an approach will also be instrumental in conserving the habitat of migratory species, such as the American redstart (*Setophaga ruticilla*)^47^, and also where species ranges span national borders, such as larger bodied mammals in the Albertine Rift, Africa^48^.

Achieving the Millennium Development Goals and post-2015 Sustainable Development Goals will require innovative solutions to complex land-use planning and policy problems^49^. An analysis of alternative futures can help visualize the outcomes of different approaches. The Inter-governmental Platform on Biodiversity and Ecosystem Services (IPBES) will also employ scenarios to address multi-scaled policy problems that encompass the natural and social sciences^50^. Through evaluation of alternative futures, we found that coordination between countries would enhance the efficiency of achieving a diverse suite of national and international policy targets, which will be relevant wherever biodiversity and industries extend across borders. Integrated planning also improves efficiency when there is variation within and between countries in the costs and opportunities for implementing policy^2^. An alternative future for the tropical forests of Borneo that captures the benefits of coordination and integrated planning could enhance both conservation and economic outcomes.

## Methods

### Land-use decision support tool

The planning goal was to meet a set of conservation and economic targets, while minimizing the opportunity cost of allocating land to particular uses (for scenarios 2–4). We used Marxan with Zones conservation planning software, which uses simulated annealing as the optimization algorithm to find multiple, near-optimal solutions for this land-use planning problem^51^. This algorithm also accounts for the impact of undesirable combinations of adjacent land uses (for example, avoids placing oil palm plantations adjacent to protected areas, where possible). Each scenario (and scenario variation) was run 1,000 times to ensure near-optimal solutions were found. We incorporated the relative probability of deforestation and assumed benefits were delivered in perpetuity (that is, if an area is re-zoned as protected, it is expected to remain forested indefinitely although we acknowledge that this may not be the case over long time frames under climate change^52^). We also discounted costs and profits in perpetuity (that is, assuming that the revenue from each land use will continue indefinitely), but did not include dynamic factors, such as commodity price fluctuations.

We accounted for the contribution to targets and opportunity costs of meeting these targets in five general land uses: (1) protected areas; (2) logging (CL or RIL, depending on scenario); (3) ITP for pulp and paper (monocultures of fast growing trees); (4) oil palm; and (5) other non-forested land uses not incorporated in the above (Supplementary Table 4). This ‘other non-forest' category represents the land remaining for other development (that is, urban, mining or other agriculture) after achieving the public policy targets. The ‘other non-forest' category was not further disaggregated or explicitly modelled due to the spatial dominance of the first four categories in the landscape. Mining, for example, while having significant localized impacts, was found to account for only a minor proportion of overall deforestation in East Kalimantan^53^. The classes of protected areas included were specific to each country. For Brunei, we accounted for forest reserves, national parks and wildlife sanctuaries. For Kalimantan, we accounted for protection forest, national parks, nature reserves, recreation/community parks and wildlife sanctuaries. In Sabah, we accounted for protection forest reserves, virgin jungle reserves, wildlife reserves, Sabah parks, wildlife sanctuaries and wildlife conservation areas. In Sarawak, we accounted for wildlife sanctuaries, national parks, protection forest, communal forest, forest reserves, hunting reserves, virgin jungle reserves and parks. We used hexagonal grids of 10 km^2^ (that is,1.7 km in-circle radius) as the base spatial unit for the analysis. We also ensured that the mean land use ‘patch' size for each solution was within ±5% of the mean of the baseline scenario (28,216 ha).

### Scenarios

*Scenario 1: baseline*. This scenario represents existing land-use allocations and is based on the following assumptions:

1. Urban and mining areas cannot be changed to other land uses.
2. All oil palm and ITP concessions are planted.
3. All areas designated for limited production or production forests become active.
4. All classes of protected areas remain protected.

The data on existing land-use allocations were compiled in accordance with Wich *et al*,^54^, including industrial oil palm plantation concession data for Kalimantan compiled by Carlson *et al*,^13^ and data for protected areas in Sabah from the Sabah Forestry Department^55^. Given the dearth of spatial information on oil palm concessions in Sabah, we assumed land classified as conversion forest would be converted to oil palm, unless another concession type was indicated. This is likely to be an overestimation of oil palm concessions in Sabah, but is appropriate for this scenario as it represents the worst case. We acknowledge that the full execution of existing land-use allocations may not be desirable due to community conflicts, low productivity and environmental issues.

*Scenario 2: state-based planning*. This scenario reflects a state-based planning approach to achieve targets (Table 2, Supplementary Table 3). The following land-use transition rules apply based on current policy or practice (Supplementary Fig. 1a):

1. Urban and mining areas cannot be changed to other land uses.
2. Current planted ITP and oil palm plantations remain.
3. All classes of protected areas remain protected.
4. New protected areas can occur where there is forest cover (that is, intact, logged, agroforest/regrowth, severely degraded).
5. New oil palm plantations can be established anywhere except urban areas, mining areas, areas not suitable for oil palm (for example,, land with a slope >45° (Supplementary Table 5b)) and planted ITP. This can include severely degraded grasslands, where suitable.
6. New ITP can be anywhere except urban areas, mining areas, areas not suitable for oil palm, oil palm concessions and planted oil palm.
7. Current oil palm concessions can only become oil palm or ‘other non-forest'.
8. Land that is not suitable for oil palm can only become ‘other non-forest', protected or logging.
9. Logging can only occur where there is sufficient forest cover (that is, not agroforest/regrowth or severely degraded forest types^56^).
10. ‘Logging' can be either CL or RIL in Sarawak and only RIL in the other states, to reflect their targets (Table 2). CL can be converted to RIL and *vice versa*.

*Scenario 3: coordinated planning within the mountainous core*. This scenario reflects the vision of the Heart of Borneo initiative, where coordinated planning between states occurs within a defined area in the mountainous interior of Borneo. Land-use transition rules within the defined Heart of Borneo area follow those stated in WWF's vision for a ‘Green Economy'^57^ including:

1. Standing primary and secondary forest cannot be developed.
2. Active logging concessions are converted to RIL.
3. Inactive logging concessions are not logged.
4. Oil palm and ITP expansion can only occur where a concession already exists and the land is degraded/idle, and excludes development in peatland, swamp forest and protected areas.
5. Urban and mining areas cannot be changed to other land uses.

As the Heart of Borneo initiative does not provide land-use transition rules beyond the defined Heart of Borneo, we have applied the land-use transition rules from scenario 2 for the remainder of the island (Supplementary Fig. 1a).

*Scenario 4: integrated planning*. This scenario reflects coordinated planning between states with the land-use transition rules employed for scenario 2, but with the following relaxations (Supplementary Fig. 1b):

1. Protected areas need not remain protected.
2. Oil palm and ITP concessions can be protected or logged where there is current forest cover (that is, intact, logged, agroforest/regrowth and severely degraded).
3. ITP can be established on oil palm concessions.
4. Oil palm and ITP concessions can become ‘other non-forest'.

This scenario (scenario 4a) was also modified to include ecosystem-based targets, representing a more integrated approach to conservation. In this modified scenario (scenario 4b), 70% of the remaining extent of each forest type (that is, montane, lowland, peat swamp, swamp, riverine, mangrove and shrubland^58^) must be protected overall. The targets for orangutan and elephant were reduced to 70% to reflect the forest type target. The aim of this was to encompass a greater range of conservation features not specifically mentioned in government policy documents, while still allowing for the expansion of other land uses.

For all scenarios, the opportunity costs were derived by discounting into perpetuity (see ‘opportunity costs' below). Similarly the expected benefits (that is, habitat for endangered species) are expected to remain in perpetuity.

### Opportunity costs

The following equation was used to determine the opportunity cost of each land-use change (adapted from Naidoo and Adamowicz^59^):


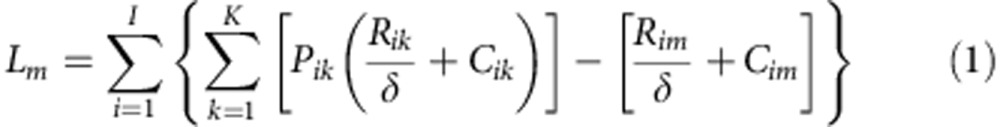


Where *L*_*m*_ is the opportunity cost of land use *m* (*L*_*m*_ is ≥0), *P*_*ik*_ is the probability that parcel *i* will be converted to land use *k*, *R*_*ik*_ is the average annual profit (or loss) associated with land use *k* for parcel *i, δ* is the discount rate, *C*_*ik*_ is the profit (or loss) from converting parcel *i* to land use *k*, *R*_*im*_ is the average annual profit from land use *m* for parcel *i* and *C*_*im*_ is the profit (or loss) from converting parcel *i* to land use *m*,

In the absence of complete information on the probability of future land use (*P*_*ik*_), we used the probability of deforestation (see online methods) and assumed that the most lucrative alternative land use would be conversion to oil palm for deforested areas or RIL for those areas that are to remain forested. Specifically, for deforested areas we used the net present value (NPV) of oil palm production (average annual oil palm profits discounted into perpetuity, plus profits from timber harvested during conversion) less the administrative costs of conversion less the NPV of the selected land use. For those areas which would remain forested, we used the NPV of RIL (annual RIL profits discounted into perpetuity, less administrative costs, less the NPV of the selected land use). For the discount rate (*δ*) we used 10%, as this is consistent with other studies in the region^60^,^61^,^62^. Further information on how the opportunity costs were calculated is provided in the Supplementary Methods.

### Targets

We analysed targets for four geopolitical units: the country of Brunei Darussalam; the two Malaysian states of Sabah and Sarawak; and Kalimantan, the Indonesian part of Borneo. We did not analyse Kalimantan at the level of provinces, because despite a process of decentralization in Indonesia, the five provinces of Kalimantan have less direct authority over their land resources compared with Brunei, Sabah and Sarawak. State governments in Sabah and Sarawak largely decide on the allocation of budgets and land uses, whereas Kalimantan depends on national level policy to inform these decisions.

The Bornean orangutan (*P. pygmaeus*), Bornean elephant (*E. maximus borneensis*) and forest cover had quantifiable governmental targets for their protection (Table 2, Supplementary Table 3). The distribution of the Bornean elephant and orangutan was determined using Maximum Entropy Modeling (MaxEnt^63^) (Fig. 1c,d). For the orangutan, this was supplemented using local knowledge, details of which can be found in Wich *et al*,^54^. For the elephant, location data (*n*=112) were collated from ground surveys and opportunistic sightings throughout the known elephant range between 1999 and 2011. We selected 11 spatial variables identified as important for determining the suitability of elephant habitat. These included: four climatic variables, precipitation annual range, precipitation seasonality, temperature annual range and temperature seasonality (WorldClim, ver. 1.4 data set; http://www.worldclim.org), road density using 1999 to 2002 Landsat digitized data^54^, soil data^64^, land cover^56^, above-ground carbon stock^65^ that was converted into Mg CO_2_ ha^−1^, and three topographic variables, elevation (WorldClim, ver. 1.4 dataset), ruggosity and slope generated from elevation data^66^. All spatial data were reclassified to 30 arc-seconds (∼1 km^2^ resolution). We set MaxEnt to measure variable importance through jack-knifing, employed the logistic output algorithm and default ‘auto feature' options. For model validation, we used cross-validation with 10 replicates^67^ and measured performance using the mean area under the receiver operating characteristic curve (AUC=0.977). Precipitation annual range, road density, soil types and temperature annual range were identified as important explanatory variables for elephant (contributing 38.2%, 16.7%, 15.1% and 9.8%, respectively). We determined a threshold probability of occurrence using the maximum sensitivity plus specificity to derive a binary map of presence/absence. This was then clipped to the known distribution of elephant within ‘forest'. Here forest was defined to include areas that have intact, logged, severely degraded logged forest or areas with forest regrowth or agroforestry (modified from 2010 SarVision data^56^: logged forests were defined as those within 5 km from a satellite-visible logging road).

### Variations

We determined if the impact of alternative interpretations of public policy targets the results, along with the impact of variations in opportunity costs (Supplementary Table 6). While the main analyses attempted to conserve all the remaining distribution of orangutan, we also considered the impact of preserving only the patches that were considered to be viable. Viable orangutan populations were determined by calculating their density in each 1 km^2^ grid cell via expert elicitation, then grouping grid cells of breeding population presence into contiguous patches (∼2,000 patches)^54^. Any of these contiguous patches that contained fewer than 250 individuals were removed, as this is considered to be the minimum viable population size for orangutan in areas with low hunting pressure^68^. We also varied the definition of ‘forest' cover, as this was not clearly specified in state government policy documents. The strict forest cover target could be met by the intact, logged or mangrove forest cover classes. The moderate and broad forest cover targets could additionally be met by the agroforest/regrowth forest class, and severely degraded logged forest could also contribute to the broad forest cover target.

We also considered the impact of assumptions about the discount rate, along with profits from oil palm, ITPs, conventional logging and RIL. We did not consider the impact of changes to once-off administrative costs or protected area management costs, as these were insignificant relative to the opportunity cost of oil palm production. We varied the profits for oil palm plantations, ITP, CL and RIL by ±50% for each land use separately and all together (Supplementary Table 6). The upper estimate for oil palm plantations was increased by 55%, to incorporate the previous peak in the fluctuations in the price of crude palm oil. We also applied a variation where the oil palm profits in Kalimantan and Sarawak matched that of Sabah, to represent a case where the management practices, environmental conditions and infrastructure is consistent across states. The cutting cycle length for both types of logging were altered by ±10 years and incorporated in the upper and lower estimates (that is, the lower logging estimate represents a 50% reduction in the profit per hectare harvested and a cutting cycle length of 40 years, while the upper logging estimate represents a 50% increase in the profit per hectare harvested and a cutting cycle length of 20 years). We varied the discount rate (of 10%) by ±5% in absence of other variations and together with the extremes of variations in profits (Supplementary Table 6).

### Classification uncertainty

To visualize the spatial uncertainty in zone allocation, we calculated the classification uncertainty (adapted from Levin *et al*,^69^):


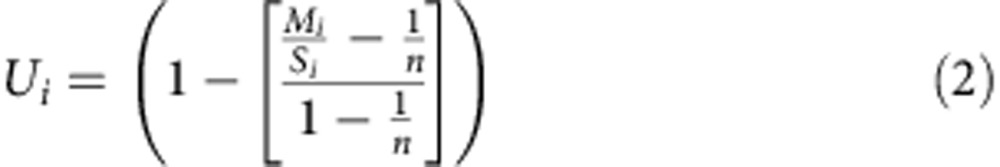


Where *U*_*i*_ is the classification uncertainty for planning unit *i*; *M*_*i*_ is the maximum set membership (the greatest number of times the planning unit was allocated to a particular zone) for planning unit *i*; *n* is the total number of zones (in this case 6) and *S*_*i*_ is the total number of runs. In this case the total number of runs was 21,000 (that is, the number of parameter variations for each scenario (21), multiplied by the number of runs per solution (1,000)). Planning units that had been allocated to each zone an equal number of times (across all the parameter variations and repetitions) would receive a value of 1, whereas planning units that had been allocated to only one zone were given a value of zero. This enabled a spatial depiction of the uncertainty, or variability, in the land use allocations for each scenario.

## Author contributions

E.M., H.P.P., J.A.W., K.A.W., N.K.A. and R.K.R. conceptualized the manuscript. E.M., F.A., J.A.W., K.A.W., M.A., M.T.G., N.K.A. and R.K.R. reviewed the literature. D.L.A.G., N.K.A., R.K.R. and S.A.W. produced the GIS data. R.K.R. ran Marxan with Zones. All authors interpreted the results and contributed to writing the paper.

## Additional information

**How to cite this article:** Runting, R. K. *et al*, Alternative futures for Borneo show the value of integrating economic and conservation targets across borders. *Nat. Commun*, 6:6819 doi: 10.1038/ncomms7819 (2015).

## Supplementary Material

Supplementary Information Supplementary Figures 1-8, Supplementary Tables 1-7, Supplementary Methods and Supplementary References

## Figures and Tables

**Table 1:** A brief description of scenarios and the socio-political challenges involved with implementing them.

Scenario name	Description	Challenges
Baseline (scenario 1)	The current land-use allocation in each state is assumed to be fully executed (for example, all oil palm concessions are planted).	Inefficient: some planned plantations are in unsuitable locations; conservation opportunities are missed.
State-based planning (scenario 2)	State or national targets are sought within each state. Minimal changes can be made to existing land-use allocations.	States must adhere to their stated targets. This may be difficult in practice due to corruption and vested interests.
Coordinated planning inside the core, with state-based planning outside (scenario 3)	Coordination between states within the mountainous interior of Borneo. State-based planning and targets are assumed outside of this area.	As per scenario 2, but all states must implement the agreed upon (but non-binding) vision of the Heart of Borneo.
Integrated planning (scenario 4a)	Uses the combined targets from scenario 2 but ignores state boundaries and modifies land-use allocations where possible.	As per scenario 2, but states must agree on island-wide targets. Implementation will require an appropriate institutional platform, and compensation mechanisms or payment schemes.
Integrated planning alternative conservation targets (scenario 4b)	As per scenario 4a, but 70% of the extant distribution of each forest type must be protected overall. The faunal targets were set at 70% of the distribution of each species to correspond to the forest cover target.	As per scenario 4a, but this scenario highlights that the current conservation targets are inadequate. Extensive consultation is required to specify island-wide conservation targets that capture a range of biodiversity features and the needs of local communities.

**Table 2:** Conservation and economic targets for Sabah, Sarawak, Kalimantan and Brunei Darussalam.

Target	Sabah, Malaysia	Sarawak, Malaysia	Kalimantan, Indonesia	Brunei Darussalam
Forest cover	50% of land area (37,000 km^2^)	50% of land area (61,885 km^2^)	45% of land area (240,587 km^2^)	75% of land area (4,337 km^2^)
Protected areas	17% of land area (12,571 km^2^)	17% of land area (21,041 km^2^)	17% of land area (90,888 km^2^)	55% of area as ‘national forest estate' (3,180 km^2^)
Orangutan	No conversion of forest with significant orangutan populations	No conversion of forest with significant orangutan populations	Stabilize all orangutan populations by 2017	NA
Elephant	Secure long-term viability of elephant populations in the state	NA	None	NA
Reduced-impact logging	All commercial forest reserve needs to be Forest Stewardship Council certified	No directive outside of the Heart of Borneo area	All production forest to be converted to reduced-impact logging	All exploitation forests follow sustainable practices
Oil palm plantations	2.1 × 10^6^ ha	2 × 10^6^ ha	Double production (to 6.9 million ‘productive hectares')	None
Industrial timber plantations	Increase by 837 km^2^ (to 1,778 km^2^)	Increase by 1,414 km^2^ (to 2,883 km^2^)	Increase by 13,900 km^2^ (to 20,186 km^2^)	None

NA, not applicable.

These data were collected by analysing public policy documents for quantifiable targets in 2012.
