# Corrigendum: Alternative futures for Borneo show the value of integrating economic and conservation targets across borders

**DOI:** 10.1038/ncomms12018

**Published:** 2016-06-14

**Authors:** Rebecca K. Runting, Erik Meijaard, Nicola K. Abram, Jessie A. Wells, David L. A. Gaveau, Marc Ancrenaz, Hugh P. Possingham, Serge A. Wich, Fitrian Ardiansyah, Melvin T. Gumal, Laurentius N. Ambu, Kerrie A. Wilson

*Nature Communications* 6: Article number:681910.1038/ncomms7819 (2015); Published 04142016; Updated 06142016

The original version of this Article contained an error in the spelling of the author Hugh P. Possingham, which was incorrectly given as Hugh P. Posssingham. This has now been corrected in both the PDF and HTML versions of the Article.

