# Supplementary material for: Alternative futures for Borneo show the value of integrating economic and conservation targets across borders
Source: Nat Commun. 2015 Apr 14;6:6819. doi: 10.1038/ncomms7819 (PMC4403346; doi:10.1038/ncomms7819)
Supplement: Supplementary Information — Supplementary Figures 1-8, Supplementary Tables 1-7, Supplementary Methods and Supplementary References [file ncomms7819-s1.pdf]

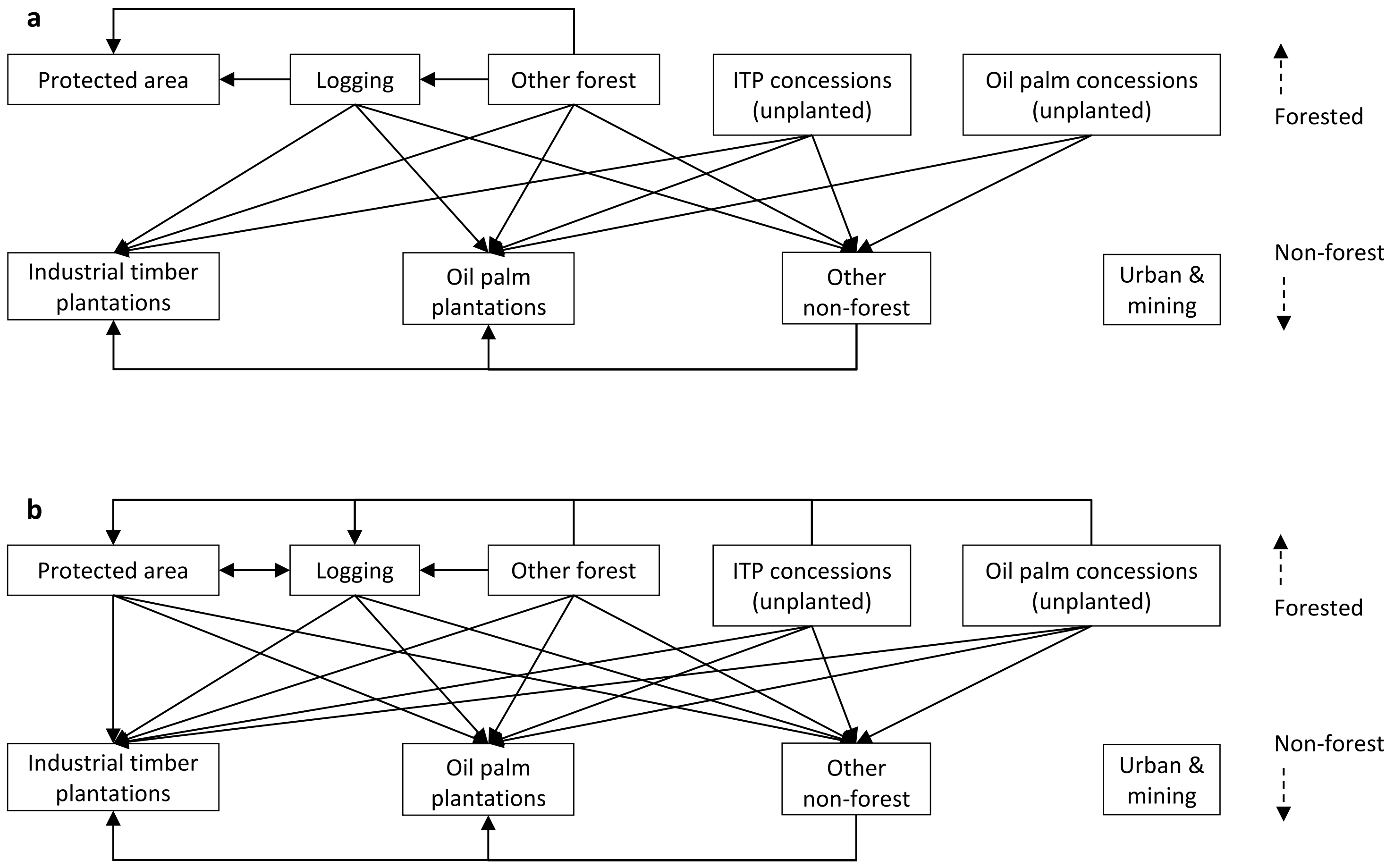

Supplementary Figure 1 | Possible land-use transitions for scenarios 2 (panel a) and 4 (panel b). Arrows show the changes in land-use allocation that are possible under each scenario (and whether uni-or bi-directional). Urban and Mining lands are not changeable, and so have no connecting arrows.

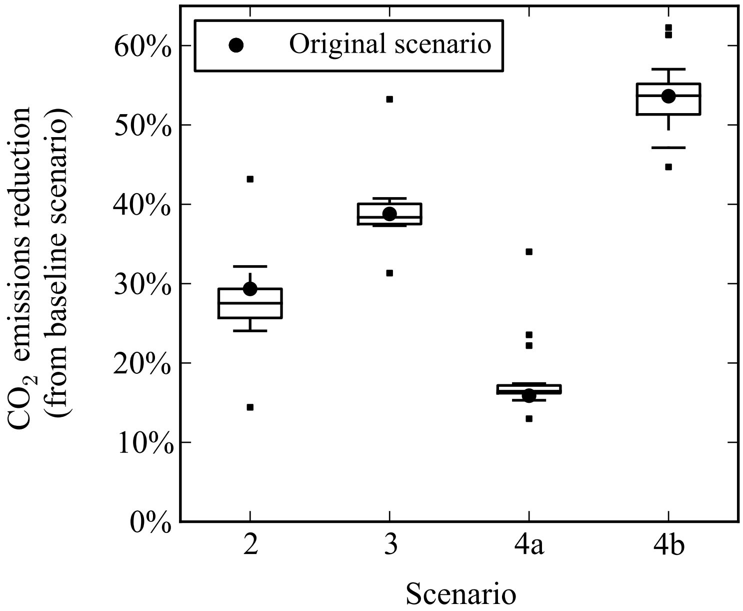

Supplementary Figure 2 | The percentage of CO_2 emissions reduction from the baseline scenario. The variations from the original scenarios were obtained by altering the economic parameters and assumptions about public policy targets.

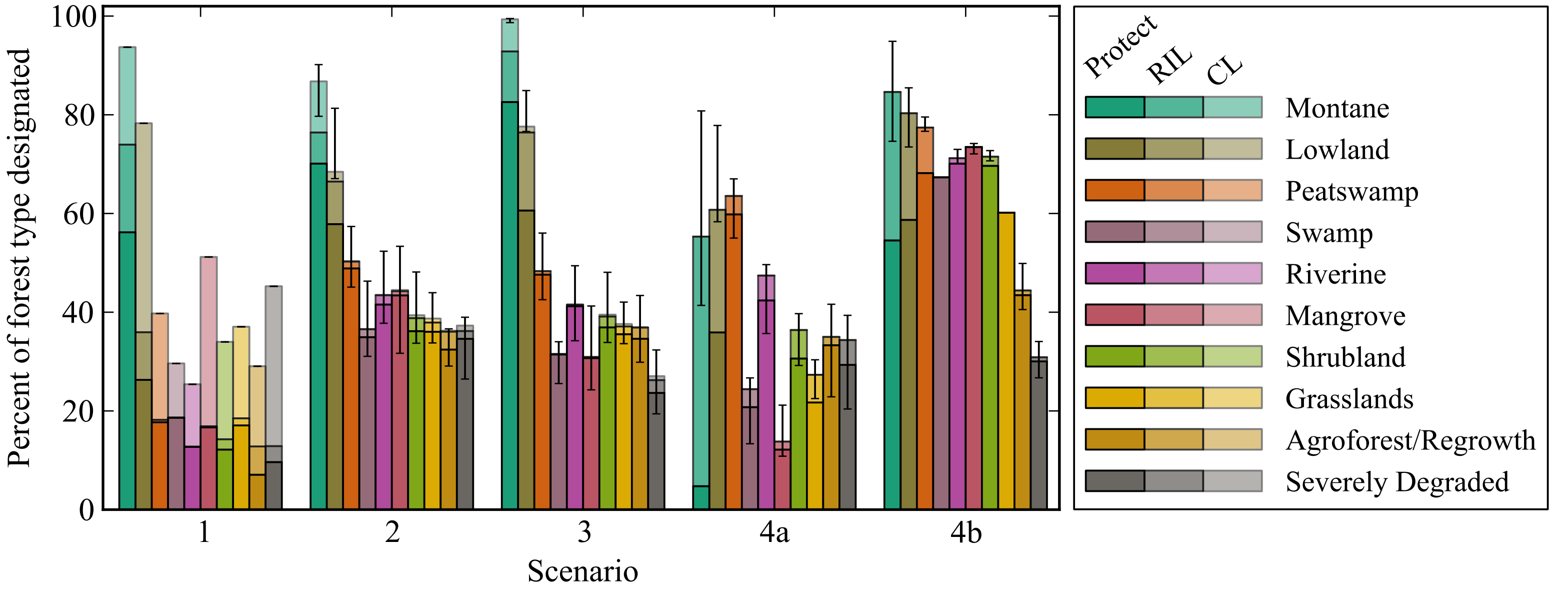

Supplementary Figure 3 | Representation of individual forest types. This shows the percentage of the extent of each forest type that is designated for protection, reduced impact logging (RIL), or conventional logging (CL). While all scenarios have a general forest cover target, this will not ensure representation of each forest type. Under scenarios 1 to 3, protected areas are concentrated in the montane forest type. Scenario 4b specifically targets each forest type individually and consequently has the most equitable representation. Forest types and extents were defined by Miettinen et al[1] for the year 2010. Error bars represent the minimum and maximum values when altering the economic parameters and assumptions about public policy targets

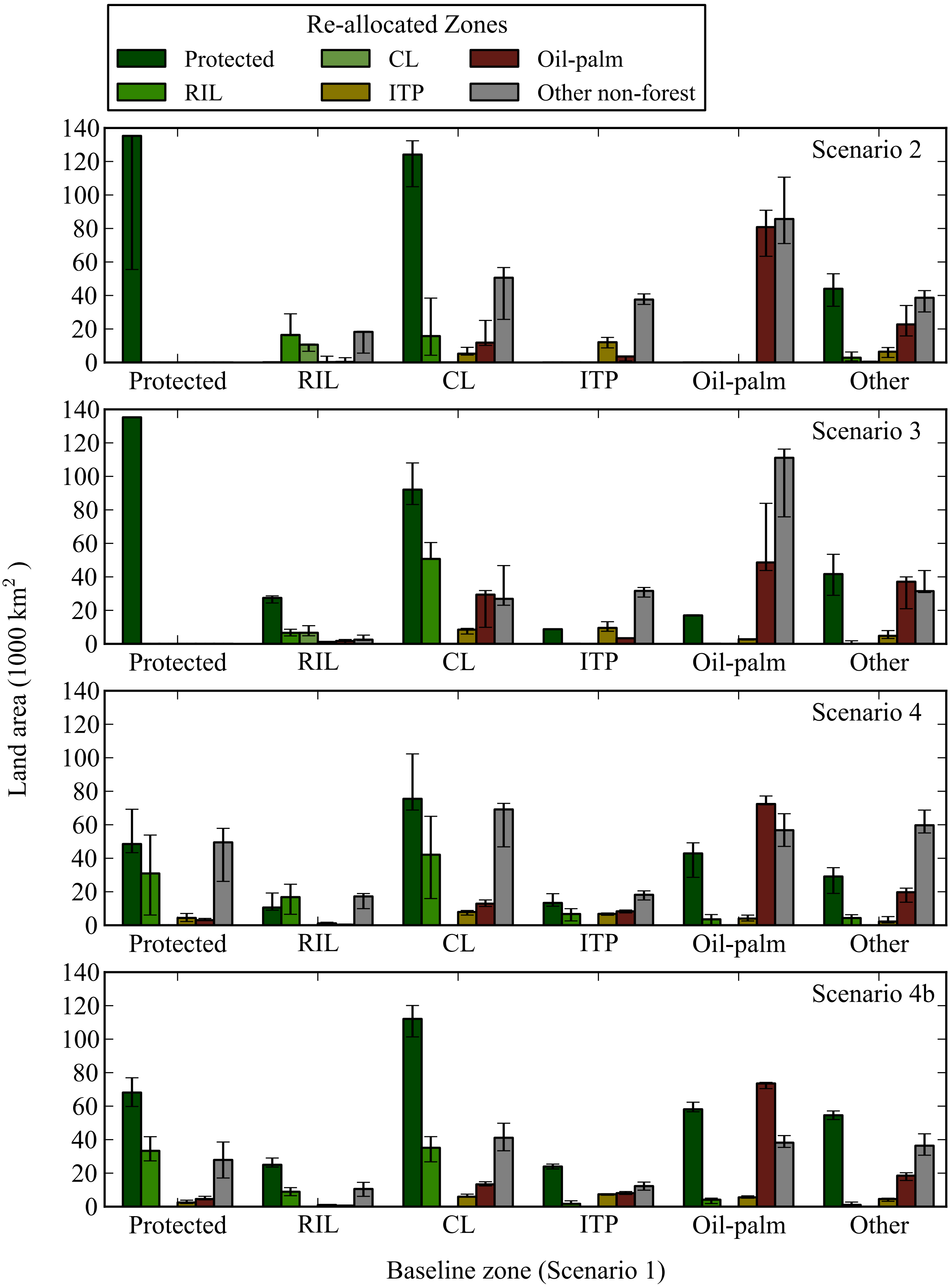

Supplementary Figure 4 | The re-allocation of land-use under different scenarios. Scenario 1 represents the existing land-use plan so was used as the baseline. Notably, there is a reallocation of protected areas in the integrated planning scenarios (4a and b). The error bars represent the minimum and maximum values when altering the economic parameters and assumptions about public policy targets.

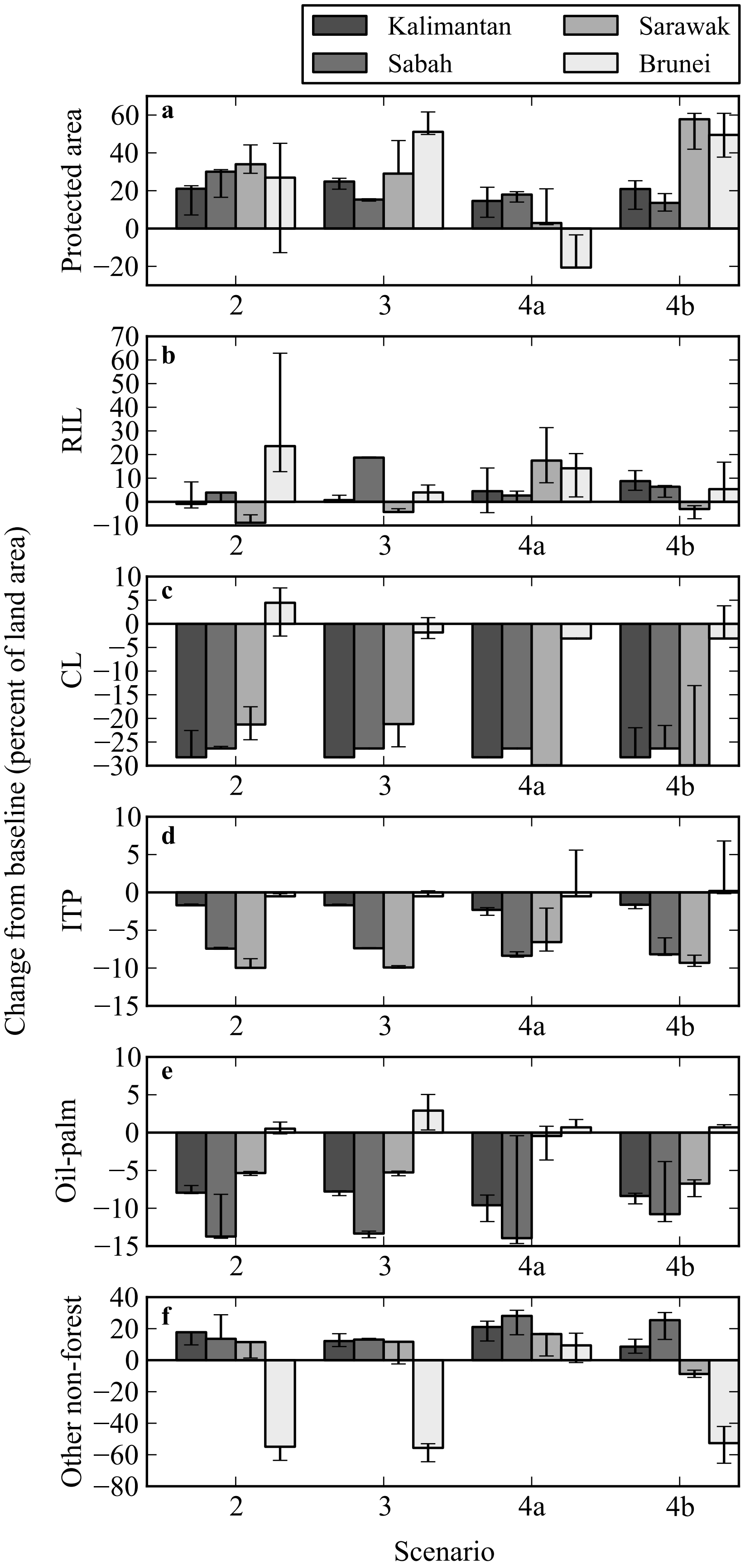

Supplementary Figure 5 | The change in the distribution of land-use zones across Bornean states when compared to the baseline scenario. This is shown for: (a) protected areas, (b) reduced impact logging (RIL), (c) conventional logging (CL), (d) industrial timber plantations (ITP), (e) oil-palm plantations, and (f) other non-forested land-uses. Error bars represent the minimum and maximum values when altering the economic parameters and assumptions about public policy targets.

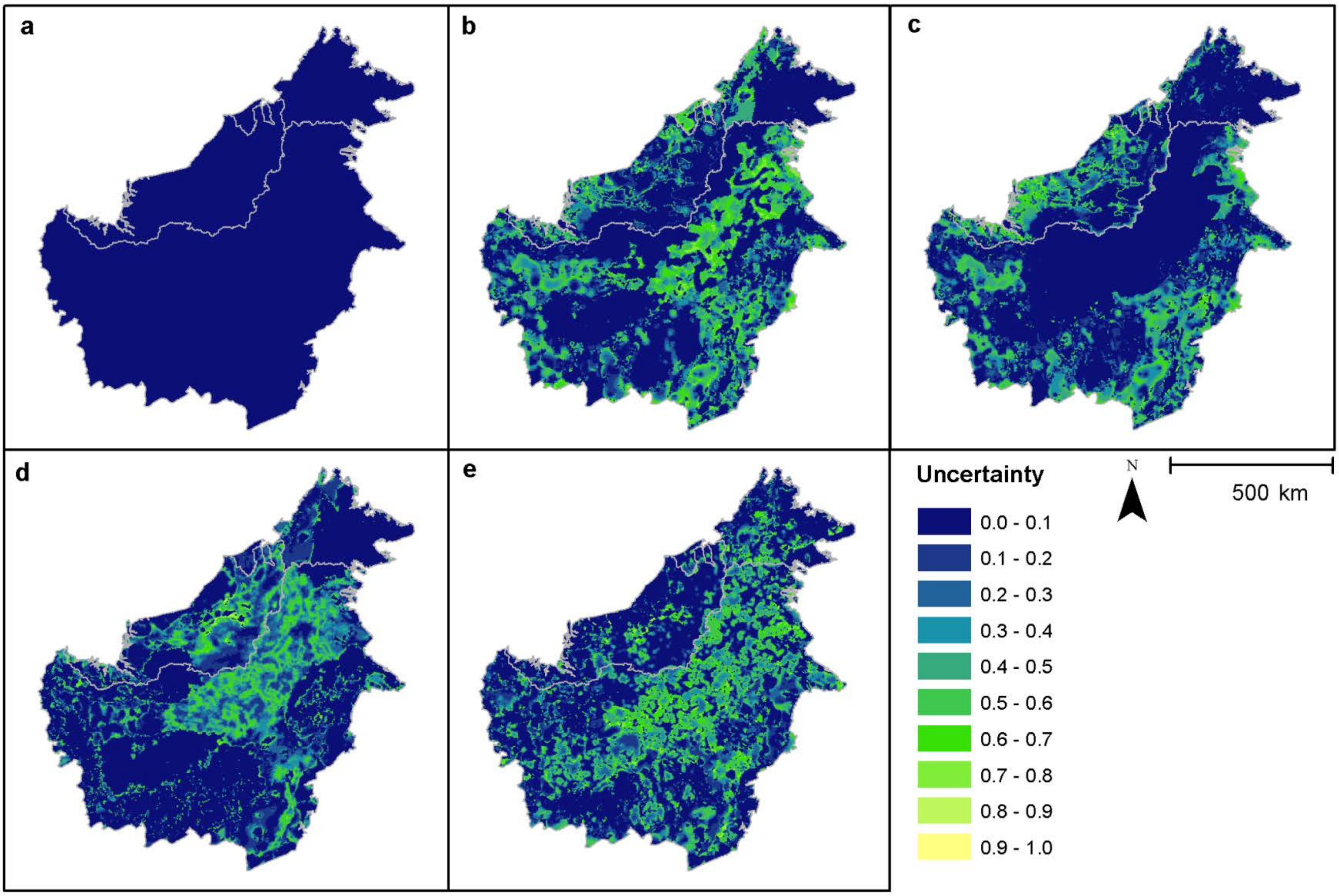

Supplementary Figure 6 | The classification uncertainty under each scenario. (a) Baseline (scenario 1), (b) state-based planning (scenario 2), (c) coordinated planning within the mountainous core, with state-based planning outside (scenario 3), (d) integrated planning with existing state targets (scenario 4a), and (e) integrated planning with alternative public policy targets for biodiversity (scenario 4b). This shows the uncertainty of allocating a planning unit to the final land-use zone. This is a combination of the classification uncertainty from multiple runs with the same input parameters, along with the variation in input parameters. There is no uncertainty surrounding zoning in scenario 1, as this scenario is based on implementing the existing land-use allocations.

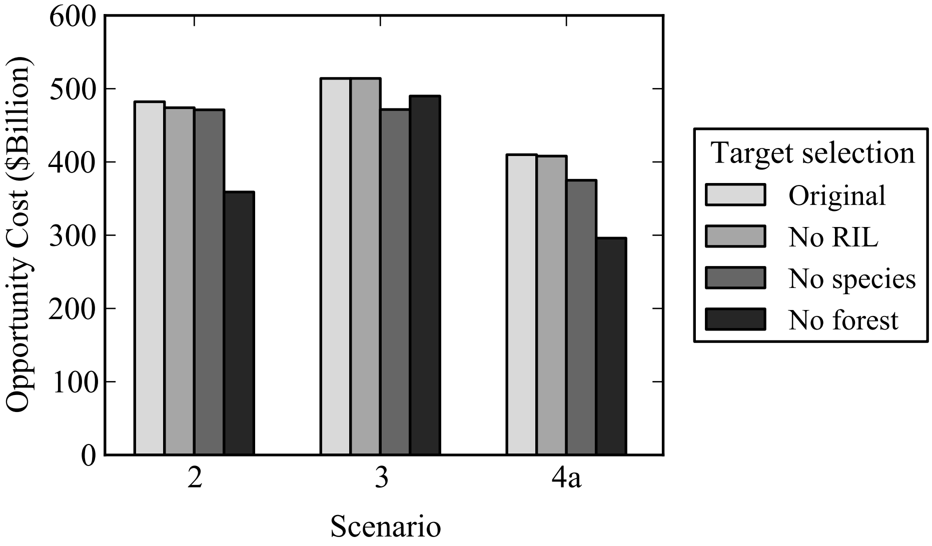

Supplementary Figure 7 | The opportunity costs across scenarios when omitting targets for reduced impact logging (RIL), species (orangutan and elephant), and forest area. Removing the requirement for RIL had only a minor reduction in the opportunity cost for each scenario, whereas removing the species or forest targets resulted in larger opportunity cost savings. Scenario 1 was not included as the land-use allocation cannot be altered, therefore changing the targets does not have an impact. Scenario 4b was also excluded, as this scenario was already a variation on the targets in Scenario 4a.

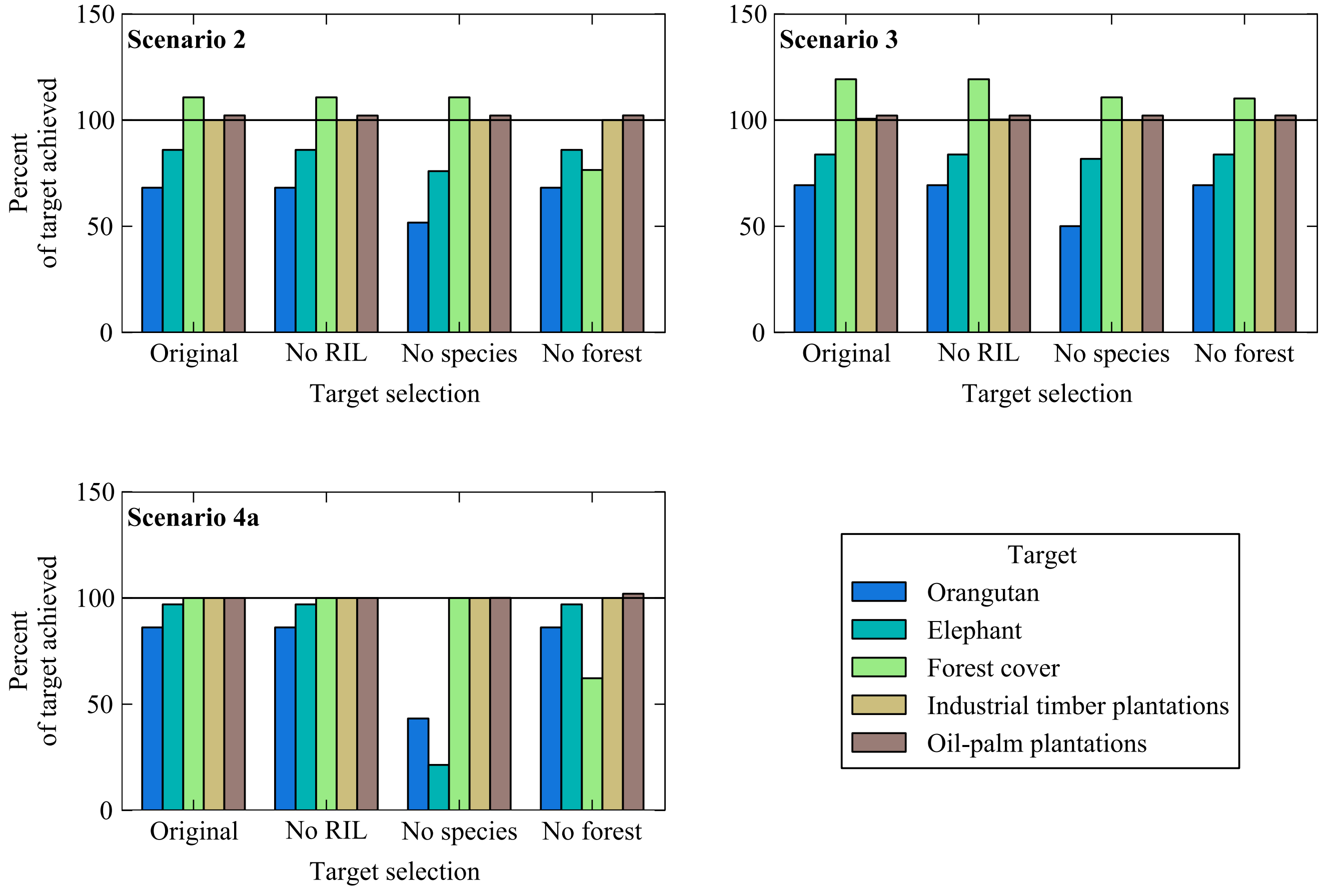

Supplementary Figure 8 | Target achievement across scenarios when omitting targets for reduced impact logging (RIL), species (orangutan and elephant) and forest area. Removing the requirement for RIL had only a negligible reduction in the target achievement for each scenario, whereas removing the species or forest targets resulted in poor conservation outcomes. Scenario 1 was not included as the land-use allocation cannot be altered, therefore changing the targets does not have an impact. Scenario 4b was also excluded, as this scenario was already a variation on the targets in Scenario 4a.

Supplementary Tables:

Supplementary Table 1 | Biological and socio-economic background for Borneo. Panel (a) shows the species occurring in Borneo, and number of endemics. Plant species counts are extrapolated estimates made by Roos et al[2]. Panel (b) shows a comparison of the three nations on Borneo across selected indicators. The corruption rank is out of the 177 countries assessed, with 1 being the least corrupt[3]. Gross domestic product (GDP) per capita is measured in purchasing power parity (PPP) equivalent to 2011US$[4].

a

Taxa	*Total number*	*# endemics*	*Source*
Plants	14,423	4,089	2
Frogs	141	88	5
Reptiles	276	89	6
Terrestrial mammals	196	40	7
Freshwater fish	394	149	8
Birds (resident and migratory)	633	53	9

b

Indicator	*Indonesia*	*Malaysia*	*Brunei*
Area on Borneo (km^2)	548,005	198,161	5,770
% of area protected	20%	9%	22%
Corruption rank[3]	114	53	38
GDP per capita (PPP)[4]	$9,561	$23,338	$71,777
Type of government	Presidential democratic republic	Constitutional elective monarchy	Absolute monarchy

Supplementary Table 2 | How the variation in input parameters changed the rankings of scenarios. Scenario are ranked by opportunity cost (1 = lowest opportunity cost). Alternative interpretations of public policy targets were not used for scenario 4b, as this scenario had already altered the public policy targets for conservation. CL and RIL refer to conventional logging and reduced impact logging respectively.

	Scenario 1	***Scenario 2***	***Scenario 3***	***Scenario 4a***	***Scenario 4b***
Original	4	2	3	1	5
Forest moderate	4	2	3	1	-
Forest strict	3	2	4	1	-
Viable orangutan	4	2	3	1	-
Low profit	4	2	3	1	5
High profit	4	2	3	1	5
Low discount rate	5	2	4	1	3
High discount rate	4	2	3	1	5
Oil-palm match Sabah	5	2	3	1	4
High oil-palm profit	5	2	3	1	4
High timber profit	4	2	3	1	5
High CL profit	4	2	3	1	5
High RIL profit	5	2	4	1	3
Low oil-palm profit	5	2	4	1	3
Low timber profit	4	2	3	1	5
Low CL profit	5	2	3	1	4
Low RIL profit	4	2	3	1	5
Low profit, low discount rate	4	2	3	1	5
Low profit, high discount rate	4	2	3	1	5
High profit, low discount rate	5	2	3	1	4
High profit, high discount rate	4	2	3	1	5

Supplementary Table 3 | Sources used to derive the public policy targets. In some cases we relied on the reporting of targets in the media due to the inaccessibility of government documents.

Target	*Sabah, Malaysia*	*Sarawak, Malaysia*	*Kalimantan, Indonesia*	*Brunei Darussalam*
Forest cover	In 1992 Malaysia pledged 50% forest cover for the country at the Rio Earth Summit	In 1992 Malaysia pledged 50% forest cover for the country at the Rio Earth Summit	Declared by the Indonesian President [10]	Declared by the Government of Brunei Darussalam [11]
Protected areas	From the Convention on Biological Diversity[12]	From the Convention on Biological Diversity[12]	From the Convention on Biological Diversity[12]	Declared by the Forestry Department[13]
Orangutan	From Sabah's Orangutan Action Plan[14]	From Sarawak's Orangutan Strategic Action Plan [15]	Declared by the Ministry of Forestry [16]	N/A
Elephant	From Sabah's Elephant Action Plan [17]	N/A	None	N/A
Reduced impact logging	Forestry director's message [18]	N/A	Declared by the Minister of Forestry and the Indonesian President[19,20]	Declared by the National Forestry Policy of Brunei Darussalam [21]
Oil-palm plantations	The Sabah Development Corridor Project states that up to 2.1 million ha of land in Sabah could be converted to agriculture[22]	Media report [23]	Media report [24,25]	None
Industrial timber plantations	Sabah's proportion of Malaysia's target of 375,000 ha by 2020[26]	Sarawak's proportion of Malaysia's target of 375,000 ha by 2020 [26]	Kalimantan's proportion of Indonesia's target of 3.6 million new hectares[27]	None

Supplementary Table 4 | The contribution of each land-use zone towards each target. CL and RIL refer to conventional logging and reduced impact logging respectively. ITP refers to industrial timber plantations.

Target / *Zone*	*Orangutan*	*Elephant*	*Forest cover*	*Protected Area*	*ITP*	*Oil-palm*
Protected	1	1	1	1	0	0
RIL	0.8	0.8	1	0	0	0
CL	0.7	0.7	1	0	0	0
ITP	0	0	0	0	1	0
Oil-palm	0	0	0	0	0	1
Other	0	0	0	0	0	0

Supplementary Table 5 | Oil-palm suitability and average annual profits. Oil-palm suitability (a) was estimated by classifying a variety of biophysical properties of land units into suitability classes for oil-palm production. The average annual profit of oil-palm (b) is separated by state and yield[28–30]. Whilst Brunei has the biophysical capacity for oil-palm, it does not currently have an oil-palm industry, so the average annual profits from neighbouring Sarawak were applied. Figures are in 2009US$ $ha^{-1}yr^{-1}$.

a

Characteristic	***1: Desirable***	***2: Minor limitations***	***3: Serious limitations***	***4: Very serious limitations***	***No data: Not at all suitable***
Slope (degree) [31]	0-12	12-16	16-24	24-45	>45 [32]
Topsoil gravel content (%) [31,33]	0-5	5-20	20-40	>40	-
Texture (USDA texture class) [31,33]	1-8	9-11	12	13	-
Drainage (class) [31,33]	4-5	3	2,6,7	1	-
Rivers (100m buffer) [34]	-	-	-	-	all
Elevation (m) [35]	< 400	400 - 500	500 - 600	600 - 1000	> 1000 or < 0
Rainfall (mm/yr) [34]	1,750–6,000	1,250–1,750		> 6,000; <1,250	

b

Suitability class	***Yield***	***Sabah***	***Sarawak***	***Kalimantan***
1	Full yield	2,545	1,704	1,496
2	25% less	1,704	1,055	897
3	50% less	840	425	317

Supplementary Table 6 | Details of which parameters were varied to determine the impact on results. CL and RIL refer to conventional logging and reduced impact logging respectively.

Variation	*Discount rate*	*Oil-palm profit*	*Industrial timber profit*	*CL profit*	*RIL profit*	*Forest cover target*	*Orangutan target*
Original	10%	-	-	-	-	Broad	All
Forest moderate	10%	-	-	-	-	Moderate	All
Forest strict	10%	-	-	-	-	Strict	All
Viable orangutan	10%	-	-	-	-	Broad	Viable
Low profit	10%	-50%	-50%	-50%	-50%	Broad	All
High profit	10%	+55%	+50%	+50%	+50%	Broad	All
Low discount rate	5%	-	-	-	-	Broad	All
High discount rate	15%	-	-	-	-	Broad	All
Oil-palm match Sabah	10%	Sabah value	-	-	-	Broad	All
High oil-palm profit	10%	+55%	-	-	-	Broad	All
High timber profit	10%	-	+50%	-	-	Broad	All
High CL profit	10%	-	-	+50%	-	Broad	All
High RIL profit	10%	-	-	-	+50%	Broad	All
Low oil-palm profit	10%	-50%	-	-	-	Broad	All
Low timber profit	10%	-	-50%	-	-	Broad	All
Low CL profit	10%	-	-	-50%	-	Broad	All
Low RIL profit	10%	-	-	-	-50%	Broad	All
Low profit, low discount rate	5%	-50%	-50%	-50%	-50%	Broad	All
Low profit, high discount rate	15%	-50%	-50%	-50%	-50%	Broad	All
High profit, low discount rate	5%	+55%	+50%	+50%	+50%	Broad	All
High profit, high discount rate	15%	+55%	+50%	+50%	+50%	Broad	All

Supplementary Table 7 | Review of estimated yields, costs, revenues and profits from logging in dipterocarp forests in Borneo. This was estimated for methods of clear-felling (CF), conventional logging (CL) or reduced impact logging (RIL). All values refer to harvested hectares, which excludes the hectares that are not harvested due to slope thresholds and RIL criteria (i.e. within a certain distance of water bodies). The cost estimates include post-landing costs and taxes. Figures are in 2009US$.

		Yield (m^3 ha^{-1})		Cost ($ m^{-3})			Revenue ($ m^{-3})		Profit ($ ha^{-1}) (Intact)			Profit ($ ha^{-1}) (Logged)		
Location	*Year published*	*CL*	*RIL*	*CL*	*RIL*	*Source*		*Source*	*CL*	*RIL*	*CF*	*CL*	*RIL*	*CF*
Sabah	*Mean*	127.75	106.00	60.36	64.20		153.00		11,835	9,230	18,635	3,503	2,732	5,263
Danum – Yayasan, Sabah [36]	2002	136.00	106.00	60.36	64.20	36,37	153.00	37	12,599	9,413	-	-	-	-
Danum, Sabah [38,39]	1992	120.00	-	60.36	-	37	153.00	37	11,117	-	-	-	-	-
Sabah [38,40]	1958	117.00	-	60.36	-	37	153.00	37	10,839	-	-	-	-	-
Sabah [38,41]	1991	138.00	-	60.36	-	37	153.00	37	12,784	-	-	-	-	-
Sarawak	*Mean*	43.70	27.80	60.03	63.73		153.00		4,063	2,484	7,782	2,194	1,341	3,718
Upper Baram, Sarawak [38,42]	2002	44.50	27.80	58.69	63.73	37,42	153.00	37	4,197	2,484	-	-	-	-
Sarawak [38,43]	1996	90.00	-	60.36	-	37	153.00	37	8,338	-	-	-	-	-
Sarawak [38,44]	1987	30.00	-	60.36	-	37	153.00	37	2,779	-	-	-	-	-
Sarawak [38,45]	1982	25.00	-	60.36	-	37	153.00	37	2,316	-	-	-	-	-
Sarawak [38,46]	1982	29.00	-	60.36	-	37	153.00	37	2,687	-	-	-	-	-
Kalimantan	*Mean*	43.61	47.83	73.87	66.20		122.00		2,100	2,679	4,033	1,134	1,447	1,927
Malinau, East Kalimantan [47]	2002	52.80	60.90	60.57	59.81	47,48	122.00	48	3,244	3,787	-	-	-	-
P.T. Limbang Ganeca, East Kalimantan [49]	2002	48.00	48.00	70.75	72.58	48,49	122.00	48	2,460	2,372	-	-	-	-
Ketapang, West Kalimantan [50]	2006	31.40	34.60	65.66	66.20	47–49	122.00	48	1,769	1,931	-	-	-	-
East Kalimantan [51]	1996	55.00	-	80.02	-	48	122.00	48	2,309	-	-	-	-	-
Central Kalimantan - 3 concessions [48]	2011	51.50	-	80.02	-	48	122.00	48	2,162	-	-	-	-	-
West Kalimantan - Suka Jaya Makmur [48]	2011	31.00	-	80.02	-	48	122.00	48	1,301	-	-	-	-	-
East Kalimantan - Balikpapan Forest Industries [48]	2011	35.60	-	80.02	-	48	122.00	48	1,494	-	-	-	-	-

Supplementary Methods:

Logging Profit

The estimated profit from timber harvesting was obtained from data on timber yields, costs and revenues for CL and RIL (Supplementary Table 6).The mean value per hexagonal 10 km^2 grid cell varied, depending on the forest condition and havestable area.

1) Forest condition. Values for forests that have been logged previously were estimated by reducing the volumes from intact forest by 46% for Kalimantan and Sarawak (based on the meta-analysis by Putz et al.[52]), and by 70.4% for Sabah (based on data from the Yayasan Sabah Forest Management Area[53]). Volumes extracted from intact forests in Sabah were generally much higher than in Kalimantan and Sarawak (c. 117-138 vs. 25-90 m^3 ha^{-1} for CL, or 106 vs. 28-48 m^3 ha^{-1} for RIL). The larger reduction factor for the volume obtainable from logged forests in Sabah partly reflects this more intense initial logging. Estimated volumes for timber from previously logged forest were much more similar across states (37.8, 23.6 and 23.5 m^3 ha^{-1} for Sabah, Sarawak and Kalimantan, respectively). Areas of open agroforests, regrowth and severely degraded burnt forests were considered unlikely to be profitable for timber extraction, due to the presence of relatively few mature trees[54].

2) *Harvestable area*. Profits are usually reported per *harvested* hectare, as distinct from all hectares in a given management unit. For CL and RIL we therefore excluded all areas with a slope greater than a threshold slope specific to the state and logging method, and for RIL we also excluded areas within specified buffering distances of water bodies or watercourses.

Slope: Within each hexagonal 10 km^2 unit, we excluded all 90 m pixels with slopes greater than a value set for RIL or CL in each state. For RIL these values were > 16.7 degrees for Kalimantan[55] and Sarawak[56], and > 25 degrees for Sabah[57]. For CL this was > 25 degrees for all states[58]. It is possible to use skyline (aerial) yarding for RIL on steeper slopes (estimated 16.7 – 35 degrees[55]), however this practice is not yet widespread and we could not find sufficient financial information on costs and yields to enable its inclusion in this study. Similarly, helicopter logging can be used on steep slopes (though damage from felling and retrieval on slopes > 25 degrees may often exceed RIL principles). However, it involves very

high costs and safety risks, and requires very tight co-ordination of felling and retrieval operations. Its use remains rare[59–61], and we found only two examples of its operation (one in Sarawak, and one in the Yayasan Sabah forest management area).

Buffering of water bodies: For RIL only, buffers of 100 m were placed around all water bodies, coastlines and large rivers (>= 50m wide)[55]. The remaining rivers in the HydroSheds dataset were buffered by 40 m[55]. The rivers in the HydroSheds dataset have minimum catchment areas of 20 km^2 [(62)], and so to allow for buffering of watercourses smaller than this threshold, we applied a uniform reduction factor of 12.2% to the remaining harvestable area in each hexagonal 10 km^2 grid cell (based on the required area for buffering small watercourses in three reserves in Sabah with moderate rainfall[63]).

The profit per hectare harvested (Supplementary Table 7) does not represent the NPV of logging. Logging companies with selective logging concessions do not harvest all of the concession area in the first year of operation, rather, a fraction of the area is harvested to ensure a continued revenue stream over the cutting cycle length[64,65]. Therefore, we divided the profit per hectare harvested by a cutting cycle length of 30 years (which is within the range of other studies[64,66–68]) to give an average annual profit per hectare. When applied to the harvestable area, this spatially explicit value represents R_i for the different types of logging in equation 1 (main text). Logging operations incurred additional costs when the area to be logged was not initially covered by a logging concession. In these cases we applied an additional, once-off cost of \$17.25 ha^{-1}, to represent official and unofficial administrative costs, based on estimates of the cost of endorsement from various levels of government.

Plantation Profit

Oil-palm suitability was estimated by classifying a variety of biophysical properties of land units into five categories based on their suitability for oil-palm production (Supplementary Table 5a). If any given pixel had at least one of the biophysical properties classed as ‘not at all suitable’, it was excluded from further analysis. The remaining pixels were summed into a cumulative suitability map, which was then tertiled into 3 suitability classes (with 1 being the most suitable). The average annual profit for oil-palm production was derived from industry specific finance models[69] based on state averages (for Sabah, Sarawak, and Kalimantan) of production per hectare of fresh fruit bundles and based on a

crude palm oil price of $800 per tonne (Supplementary Table 5b). Different scenarios of yield (full yield, 25% less, and 50% less) were applied to the 3 suitability classes to produce a Borneo-wide layer of potential revenue from oil-palm production (which was summarised at the planning unit level and used as R_i for oil-palm in equation 1). Oil palm is particularly well adapted to the humid tropics, which combined with growing demand, means revenues are likely to continue well into the future[70]. Oil-palm production was therefore measured in productive hectare equivalents (i.e. one hectare of oil-palm planted on land with 50% productive capacity equates to half a hectare of oil-palm production).

The average annual profit of industrial timber plantations (adjusted to 2009 US$) was based on estimates from the Indonesian Forest Climate Alliance[71]. This attributed a different average annual profit to mineral ($283.04) and peat ($177.08) soils, due to the difference in productivity of these soil types. Any areas that were 'not at all suitable' for oil-palm were also considered to be unsuitable for ITP and were excluded from the calculation. The final values were summarised at the planning unit level and used as R_i for ITP in equation 1.

An additional, once-off cost (in year 0) was attributable in the cases where plantations were allocated on land that does not currently have a relevant concession (allowable in scenarios 2-4). For oil-palm, there are many steps involved in obtaining a licence. As official figures were unavailable, we estimated this value at $907.58 ha^{-1} (2009 US$) using unofficial sources[72]. For industrial timber plantations we estimated this value at $154 ha^{-1} based on official guidelines[73].

In addition to revenues from oil-palm or industrial timber production, significant additional revenue can arise from timber harvest during conversion from forest to plantations[74]. This was a once-off profit attributable to year 0 (i.e. it was not discounted). Timber revenues from clear-felling before conversion to oil-palm were estimated from logging revenues for each state and forest type (intact or previously logged), as given in the description of timber harvesting profits, combined with estimates of the percentage of additional timber that could be obtained from clear-felling rather than selective logging (Supplementary Table 7). The multiplication factors were estimated from data on timber harvesting profits (revenues minus costs) from three rounds of logging in the Yayasan Sabah Forest Management Area (from an area of approximately 310,000 ha)[53]. That study reported values from logging in intact forests, from logging in previously logged forests, and from

clear-felling of twice-logged forests. We assumed that the total volumes attainable by clear-felling an intact forest, or a logged forest, would be similar to the sum of volumes from sequential logging rounds reported in that study. For example, for intact forests, we assumed the amount that could be clear-felled in a single cut is similar to the sum of volumes reported from the first and second selective logging events, and the final clear-felling of the remnant stand. This calculation also assumes that levels of damage or wastage would be similar whether the felling occurs in sequential rounds or as a single clear-cut. It also does not account for possible regeneration between logging events, although this may have been small given the lengths of time between rotations in the Yayasan Sabah Forest Management Area (mean 16 years from first to second cut, and 1-7 years from second cut to clear-felling)[53]. For Sarawak and Kalimantan, we modified the selective logging to clear-felling ratios to account for the higher relative volumes remaining after each logging round in these states (yields from logged forests being approximately 54% of yields from intact forest, compared to approximately 28% in Sabah). These clear-felling profits, less administrative start-up costs, form C_i for oil-palm or ITP in equation 1.

Protected area costs

The average annual management costs for protected areas (per hectare) was based on the optimal management of large Indonesian terrestrial national parks (approx. 120,000 ha)[75]. This value (of 2004 US\$6.17 ha^{-1} yr^{-1}) was similar to other estimates (e.g. Wilson et al.[76,77]) and was adjusted to 2009 US\$ (\$7.01 $ha^{-1}yr^{-1}$) . The estimate includes field and administrative staff, equipment and infrastructure maintenance[75]. This 'loss' forms R_i for protected areas in equation 1. Additional start-up costs arise when a new protected area is established, which was estimated at \$50 ha^{-1}[(76)]. Were applicable -\$50 ha^{-1} forms C_i for protected areas in equation 1.

Probability of deforestation

We employed a tree cover loss map for the period 2000–2010 (60x60m grid cell size) as the base dataset for modelling the probability of deforestation[78,79]. In this dataset ‘tree cover’ is defined as areas of trees (≥5m height) with >25% canopy cover and ‘tree cover loss’ as the removal of tree stands. We restricted our analysis to losses of intact forest cover that existed in year 2000. We randomly sampled 3,391 cells (of 6,234 available at a 1 km^2

resolution) and, of these, 451 cells had lost at least 20 hectares of forest. An equal number of cells with no forest loss were also randomly selected. The sub-sample of 902 cells was analysed using logistic regression, with elevation[80], distance to cities (cities were defined as having a constructed surface area density greater than two per cent, using data from Sutton et al.[81]), soil type (peat or mineral), and land-use (protected area, logging concession, limited production forest, production forest, conversion forest, monoculture industrial timber plantation or oil-palm plantation concession[32,82]) employed as explanatory variables. The final model (R^2 of 0.68) included elevation and land-use as the most significant explanatory variables ($p < 0.05$), with forest at low elevations, in oil-palm plantation concessions and with conversion forest status having the strongest relationship with areas that have been cleared. The spatial layers of each of these variables were weighted by their respective coefficient to produce a relative probability map of deforestation.

Carbon

We evaluated the change in carbon stock for each scenario relative to the current land-use plan (scenario 1). We calculated potential CO_2 emissions as the difference in time averaged CO_2 relative to a simple baseline scenario in which any area of existing forest is converted to oil-palm. Emissions from this conversion are assumed to equate to the extant aboveground carbon[83] and including peat carbon if on peat soil. Carbon was converted to CO_2e using an emissions factor of 3.67[84,85]. Peat soil carbon net emissions were estimated using net CO_2 fluxes for a 25-year period[86], which considers all inputs and outputs (and a single fire during forest clearance), giving an estimate of 1503 Mg CO_2e ha^{-1} over a 25-year time horizon. Below-ground carbon was not considered for mineral soils, due to a lack of data for all land-use transitions, and the comparatively small changes in time-averaged carbon stocks on most mineral soil types (e.g. converting primary forest to oil-palm would emit 32.0 Mg CO_2e ha^{-1} over 49 years on mineral soils[87], compared to 1503 Mg CO_2e ha^{-1} for the same conversion over 25 years on peat soils).

We assumed protected areas would retain extant aboveground and peat carbon, and sequester carbon through natural regeneration. For degraded forest and forest regrowth with extant aboveground carbon contents less than intact forest, we assumed regeneration would increase aboveground carbon stocks to equal that of the average for intact forest. For severely degraded logged forests, we assumed protection would only increase the stock of carbon by 5%. Most of this class is in East Kalimantan Province and these forests were severely burned

twice, in March-April 1983 and March-April 1998 (i.e. during the two most intense El Niño fire pulses on record, also declared national disasters in Indonesia[88]). Because of further burning, these areas have exhibited limited natural regeneration, showing high levels of cover by invasive grass species, and are unlikely to regain significant quantities of forest cover or biomass without active restoration[89]. Active restoration was not considered in these analyses (i.e. we assumed no carbon benefits from protection of lands that currently have no forest cover).

RIL was assumed to result in a reduction of 30% of above ground carbon, relative to intact forest, and CL a reduction of 60%[32], relative to intact forest. CL was also assumed to emit approximately 347.5 Mg CO_2 ha^{-1} if on peat soils due to soil disturbance[86]. Plantations (for industrial timber or oil-palm) were assigned no net change when planted on non-forest areas (0 Mg CO_2 ha^{-1}), because the carbon sequestered in industrial timber and oil-palm plantations is ultimately released when trees are harvested. For the "other non-forest" land-use class, we assumed worst case carbon emissions (i.e. that of oil-palm).

Supplementary References

1. Miettinen, J., Shi, C., Tan, W. J. & Liew, S. C. 2010 land cover map of insular Southeast Asia in 250-m spatial resolution. *Remote Sens. Lett.* **3**, 11–20 (2012).

2. Roos, M. C., Kessler, P. J. A., Robbert Gradstein, S. & Baas, P. Species diversity and endemism of five major Malesian islands: diversity-area relationships. *J Biogeogr.* **31**, 1893–1908 (2004).

3. Transparency International. *Corruption perceptions index 2013*. 8 (Transparency International: Berlin, Germany, 2013).

4. The World Bank Group. GDP per capita, PPP (current international $). http://data.worldbank.org/indicator/NY.GDP.PCAP.PP.CD?order=wbapi_data_value_2013 wbapi_data_value wbapi_data_value-last&sort=desc (2015).

5. Inger, R. F. & Voris, H. K. The biogeographical relations of the frogs and snakes of Sundaland. *J Biogeogr.* **28**, 863–891 (2001).

6. Uetz, P. & Hošek, J. (eds.) The Reptile Database. http://www.reptile-database.org (2013).

7. Corbet, G. B. & Hil, J. E. *The mammals of the Indomalayan region: a systematic review*. (Oxford University Press: Oxford, United Kingdom, 1992).

8. Kottelat, M. Zoogeography of the fishes from Indochinese inland waters with an annotated check list. *Bulletin Zoölogisch Museum, Universiteit van Amsterdam* **12**, 1–54 (1989).

9. Myres, S. *A field guide to the Birds of Borneo*. (New Holland Publishers: London, Cape Town, Sydney, Auckland, 2009).

10. President of the Republic of Indonesia. *Peraturn Presiden Republik Indonesia, Nomor 3 Tahun 2012, Tentang, Rencana Tata Ruang Pulau Kalimantan*. (Jakarta, Indonesia, 2012).

11. Government of Brunei Darussalam. *4th National Report. Convention on Biological Diversity*. (2008).

12. CBD. COP 10 Decision X/2: Strategic Plan for Biodiversity 2011–2020. www.cbd.int/decision/cop/?id=12268 (2010).

13. Government of Brunei Darussalam Forestry Department. *National Forest Policy*. (Ministry of Industry and Primary Resources: 1989).

14. Sabah Wildlife Department. *Orangutan Action Plan 2012 - 2016*. (Kota Kinabalu, Sabah, Malaysia, 2011).

15. Gumal, M. & Tisen, O. B. *Orangutan Strategic Action Plan: Trans-Boundary Biodiversity Conservation Area*. 85 (ITTO-SFD: Kuching, Sarawak, 2010).

16. Soehartono, T. *et al. Strategi dan rencana aksi konservasi orangutan Indonesia 2007-2017*. (Ministry of Forestry of the Republic of Indonesia: Jakarta, Indonesia, 2007).

17. Sabah Wildlife Department. *Elephant Action Plan (2012 - 2016)*. (Kota Kinabalu, Sabah, Malaysia, 2011).

18. Mannan, D. S. *Director's Message*. (Kota Kinabalu, Sabah, Malaysia, 2012).

19. Ministry of Forestry of the Republic of Indonesia. *Keputusan Menteri Kehutanan Nomor: 4795/Kpts-II/2002 tentang Kriteria dan Indikator Pengelolaan Hutan Alam Produksi Lestari Pada Unit Pengelolaan*. (Jakarta, Indonesia, 2002).

20. President of the Republic of Indonesia. *Peraturan Pemerintah Republik Indonesia Nomor 6 Tahun 2007 Tentang Tata Hutan dan Penyusunan Rencana Pengelolaan Hutan, Serta Pemanfaatan Hutan*. (Jakarta, Indonesia, 2007).

21. Brunei Forestry Department. *National Forestry Policy of Brunei Darussalam*. (Bandar Seri Begawan, Brunei Darulssalam, 2012).

22. Sabah Economic Development and Investment Authority. Chapter 3: Sustainability is the foundation for growth. *Sabah Development Corridor* (2008).

23. Oil palm acreage target achievable. *The Borneo Post* (2012).

24. Bahroeny, J. J. Palm oil as an economic pillar of Indonesia. *Jakarta Post* (2009).

25. Gilbert, N. Palm-oil boom raises conservation concerns. Industry urged towards sustainable farming practices as rising demand drives deforestation. *Nature News* (2012).

26. Malaysian Timber Industry Board. Development of Forest Plantation Programme. http://www.mtib.gov.my/index.php?option=com_content&view=article&id=94:forest-plantation&catid=212:forest-plantation&Itemid=130&lang=en (2009).

27. Obidzinski, K. & Dermawan, A. Smallholder timber plantation development in Indonesia: what is preventing progress? *Int. For. Rev.* **12**, 339–348 (2010).

28. Direktorat Jenderal Perkebunan. *Statistik Perkebunan Indonesia 2009-2011*. (Jakarta, Indonesia, 2012).

29. MPOB. *Palm oil cost of production Malaysia 2008. A report of the MPOB Palm Oil Cost of Production Survey 2009*. (Techno-Economics Unit, Economics and Industry Development Division, Malaysian Palm Oil Board: Kota Kinabalu, Malaysia, 2009).

30. MPOB. *Malaysian Oil Palm Statistics 2011*. (Malaysian Palm Oil Board, Ministry of Plantation Industries and Commodities: Kota Kinabalu, Malaysia, 2012).

31. Applied Agricultural Resources Sdn Bhd. Soil Requirements of Plantation Tree Crops. http://www.aarsb.com.my/AgroMgmt/OilPalm/SoilMgmt/General/Requirement.html (2012).

32. Carlson, K. M. *et al.* Committed carbon emissions, deforestation, and community land conversion from oil palm plantation expansion in West Kalimantan, Indonesia. *Proc. Natl. Acad. Sci. U.S.A.* **109**, 7559–7564 (2012).

33. FAO/IIASA/ISRIC/ISSCAS/JRC *Harmonized World Soil Database (version 1.2).* (FAO and Lazenburg, Austria: IIASA: Rome, Italy, 2012).

34. Gingold, B. *et al. How to identify degraded land for sustainable palm oil in Indonesia. Working Paper*. (World Resources Institute and Sekala: Washington D.C., 2012).

35. Mantel, S., Wösten, H. & Verhagen, J. *Biophysical Land Suitability for Oil Palm in Kalimantan, Indonesia. Report 2007/01.* (ISRIC - World Soil Information, Alterra, Plant Research International, Wageningen UR: Wageningen, 2007).

36. Tay, J., Healey, J. & Price, C. Financial assessment of reduced impact logging techniques in Sabah, Malaysia. *Applying Reduced Impact Logging to Advance Sustainable Forest Management International Conference Proceedings. Kuching, Malaysia:* (2002).

37. Fisher, B., Edwards, D., Giam, X. & Wilcove, D. The high costs of conserving Southeast Asia's lowland rainforests. *Front. Ecol. Environ.* **9**, 329–334 (2011).

38. Edwards, D., Fisher, B., Giam, X. & Wilcove, D. Underestimating the costs of conservation in Southeast Asia. *Front. Ecol. Environ.* **9**, 544–545 (2011).

39. Marsh, C. & Greer, A. Forest land-use in Sabah, Malaysia – an introduction to Danum Valley. *Philos. Trans. R. Soc. London Ser. B* **335**, 331–39 (1992).

40. Nicholson, D. An analysis of logging damage in tropical rain forest, North Borneo. *Malaysian Forester* **21**, 235–45 (1958).

41. Sim, B. & Nykvist, N. Impact of forest harvesting and replanting. *J. Trop. For. Sci.* **3**, 251–84 (1991).

42. Richter, F. *Financial and economic assessment of timber harvesting operations in Sarawak, Malaysia. Forest Harvesting Case Study 17.* (Food and Agriculture Organization of the United Nations: Rome, Italy, 2002).

43. Grieser-Johns, A. Bird population persistence in Sabahan logging concessions. *Biol. Conserv.* **75**, 3–10 (1996).

44. Hutchinson, I. Improvement thinning in natural tropical forests: aspects and institutionalization. *Natural management of tropical moist forests – silvicultural and management prospects of sustained utilization* (1987).

45. Lee, H. The development of silvicultural systems in the hill forests of Malaysia. *Malaysian Forester* **45**, 1–9 (1982).

46. Mattsson-Marn, H. *Forestry development project Sarawak, Malaysia. The planning and design of the forest harvesting and log transport operation in the mixed*

dipterocarp forest of Sarawak. Field Doc No 17. (Food and Agriculture Organization of the United Nations: Rome, Italy, 1982).

47. Dwiprabowo, H., Grulois, S., Sist, P. & Kartawinata, K. *Cost–benefit analysis of reduced-impact logging in a lowland Dipterocarp forest of Malinau, East Kalimantan*. (CIFOR and ITTO: Bogor, Indonesia, 2002).

48. Ruslandi, Venter, O. & Putz, F. E. Overestimating conservation costs in Southeast Asia. *Front. Ecol. Environ.* **9**, 542–543 (2011).

49. Hinrichs, A. *et al.* Simple measures with substantial impact: implementing RIL in one forest concession in East Kalimantan. *Applying Reduced Impact Logging to Advance Sustainable Forest Management. International conference proceedings 26 February to 1 March 2001, Kuching, Malaysia* (2002).

50. Elias, I. Financial Analysis of RIL Implementation in the Forest Concession Area of PT. Suka Jaya Makmur, West Kalimantan and Its Future Implementation Options, by the Faculty of Forestry, Bogor Agricultural University. *ITTO MoF Regional Workshop - RIL Implementation in Indonesia with reference to Asia-Pacific region: Review and experiences* 169–182 (2006).

51. Muladi, S. Quantification and use of dipterocarp wood residue in east Kalimantan. *Dipterocarp forest ecosystems: towards sustainable management* (1996).

52. Putz, F. E. *et al.* Sustaining conservation values in selectively logged tropical forests: the attained and the attainable. *Conserv. Lett.* **5**, 296–303 (2012).

53. Fisher, B. *et al.* Cost-effective conservation: calculating biodiversity and logging trade-offs in Southeast Asia. *Conserv. Lett.* **4**, 443–450 (2011).

54. Slik, J. W. F., Verburg, R. W. & Keßler, P. J. . Effects of fire and selective logging on the tree species composition of lowland dipterocarp forest in East Kalimantan, Indonesia. *Biodivers. Conserv.* **11**, 85–98 (2002).

55. Sist, P., Dykstra, D. & Fimbel, R. *Reduced-impact logging guidelines for lowland and hill dipterocarp forests in Indonesia. Occasional Paper No. 15*. 19 (CIFOR: Bogor, Indonesia, 1998).

56. Richter, F. *Financial and economic assessment of timber harvesting operations in Sarawak, Malaysia. Forest Harvesting Case Study 17*. (Food and Agriculture Organization of the United Nations: Rome, 2002).

57. Lohuji, P. L. & Taumas, R. *RIL Operation Guide Book Specifically for Tracked Skidder Use*. (Sabah Forestry Department: Sandakan, Sabah, Malaysia, 1998).

58. ECD. *Environmental Impact Assessment (EIA) Guidelines for logging and forest clearance activities*. 87 (State Environmental Conservation Department (ECD): Sabah, Malaysia, 2002).

59. Asia-Pacific Forestry Comission. *Taking stock: Assessing progress in developing and implementing codes of practice for forest harvesting in ASEAN member countries*. (ASEAN - Association of South East Asian Nations and FAO - Food and Agriculture Organization of the United Nations: Jakarta, Indonesia, 2006).

60. Bryan, J. E. *et al.* Extreme Differences in Forest Degradation in Borneo: Comparing Practices in Sarawak, Sabah, and Brunei. *PLOS ONE* **8**, e69679 (2013).

61. Thang, H. C. & Chappell, N. A. Minimising the hydrological impact of forest harvesting in Malaysia's rain forests. *Forests, Water and People in the Humid Tropics* 853–866 (2005).

62. Lehner, B., Verdin, K. & Jarvis, A. *HydroSHEDS Technical Documentation*. (World Wildlife Fund US: Washington, DC, 2006).

63. Pinard, M. A., Putz, F. E. & Tay, J. Lessons learned from the implementation of reduced-impact logging in hilly terrain in Sabah, Malaysia. *Int. For. Rev.* **2**, 33–39 (2000).

64. Sabah Forestry Department. *Deramakot Forest Reserve (FMU 19A) Mid term review: 2nd forest management plan (2005-2014)*. (Sabah Forestry Department: Sandakan, Sabah, Malaysia, 2009).

65. Edwards, D. P., Tobias, J. A., Sheil, D., Meijaard, E. & Laurance, W. F. Maintaining ecosystem function and services in logged tropical forests. *Trends Ecol. Evol.* **29**, 511–520 (2014).

66. Van Gardingen, P. *et al.* Financial and ecological analysis of management options for logged-over Dipterocarp forests in Indonesian Borneo. *For. Ecol. Manage.* **183**, 1–29 (2003).

67. Fisher, B., Edwards, D., Giam, X. & Wilcove, D. The high costs of conserving Southeast Asia's lowland rainforests. *Front. Ecol. Environ.* **9**, 329–334 (2011).

68. Bryan, J. E. *et al.* Extreme Differences in Forest Degradation in Borneo: Comparing Practices in Sarawak, Sabah, and Brunei. *PLOS ONE* **8**, e69679 (2013).

69. Abram, N. K. *et al.* Synergies for improving oil palm production and forest conservation in floodplain landscapes. *PLOS ONE* **9,** e95388 (2014).

70. Villoria, N. B., Golub, A., Byerlee, D. & Stevenson, J. Will Yield Improvements on the Forest Frontier Reduce Greenhouse Gas Emissions? A Global Analysis of Oil Palm. *Am. J. Agric. Econ.* **95**, 1301–1308 (2013).

71. Indonesian Forest Climate Alliance. *Indonesian Forest Climate Alliance Consolidation Report. Reducing Emissions from Deforestation and Degradation in Indonesia*. 218 (Jakarta, Indonesia, 2008).

72. Borneo Climate Change. Uang Bawah Tangan Pengurusan Izin Perkebunan. http://borneoclimatechange.org/berita-641-uang-bawah-tangan-pengurusan-izin-perkebunan.html (2013).

73. Republik Indonesia. *Peraturan Menteri Kehutanan Republik Indonesia. Nomor: P.26/Menhut-II/2009*. (Republik Indonesia: Jakarta, Indonesia, 2009).

74. Venter, O. *et al.* Harnessing carbon payments to protect biodiversity. *Science* **326**, 1368 (2009).

75. McQuistan, C. I., Fahmi, Z., Leisher, C., Halim, A. & Adi, S. W. *Protected Area Funding in Indonesia*. 30 (State Ministry of Environment Republic of Indonesia: Jakarta, Indonesia, 2006).

76. Wilson, K. A. *et al.* Conserving biodiversity in production landscapes. *Ecol. Appl.* **20**, 1721–1732 (2010).

77. Kementrian Kehutanan *Rencana Kerja. Direktorat Jenderal PHKA. Tahun 2014 (Annual Workplan 2014)*. (Jakarta, Indonesia, 2013).

78. Broich, M. *et al.* Time-series analysis of multi-resolution optical imagery for quantifying forest cover loss in Sumatra and Kalimantan, Indonesia. *Int. J. Appl. Earth Obs. Geoinf.* **13**, 277–291 (2011).

79. Hansen, M. C. *et al.* Humid tropical forest clearing from 2000 to 2005 quantified by using multitemporal and multiresolution remotely sensed data. *Proc. Natl. Acad. Sci. U.S.A.* **105**, 9439–44 (2008).

80. Rabus, B., Eineder, M., Roth, A. & Bamler, R. The shuttle radar topography mission—a new class of digital elevation models acquired by spaceborne radar. *ISPRS J. Photogramm. Remote Sens.* **57**, 241–262 (2003).

81. Sutton, P. *et al. Impervious Surface Area of South East Asia*. (National Geophysical Data Centre, National Oceanic and Atmospheric Administration: Boulder, Colorado USA, 2010).

82. Wich, S. A. *et al.* Understanding the impacts of land-use policies on a threatened species: is there a future for the Bornean orang-utan? *PLOS ONE* **7**, e49142 (2012).

83. Baccini, A. *et al.* Estimated carbon dioxide emissions from tropical deforestation improved by carbon-density maps. *Nat. Clim. Chang.* **2**, 182–185 (2012).

84. Pendleton, L. *et al.* Estimating global “blue carbon” emissions from conversion and degradation of vegetated coastal ecosystems. *PLOS ONE* **7**, e43542 (2012).

85. IPCC. *Good Practice Guidance for Land use, Land-use Change, and Forestry*. (Institute for Global Environmental Strategies: Kamiyamaguchi, Japan, 2006).

86. Hergoualc'h, K. & Verchot, L. Rationalizing peat greenhouse gases emissions from land use and land-use change in Southeast Asia. *2013 Wetlands Supplement to the 2006 IPCC guidelines for national greenhouse gas inventories* (2013).

87. Don, A., Schumacher, J. & Freibauer, A. Impact of tropical land-use change on soil organic carbon stocks - a meta-analysis. *Glob. Chang. Biol.* **17**, 1658–1670 (2011).

88. Dennis, R. A. *et al.* Fire, People and Pixels: Linking Social Science and Remote Sensing to Understand Underlying Causes and Impact of Fires in Indonesia. *Hum. Ecol.* **33**, 465–504 (2005).

89. Kartawinata, K. A wider view of the fire hazard. *Southeast Asia's environmental future: the search for sustainability* 261–266 (1993).
